# Extracellular vesicles enriched with miR-486 from Tetramethylpyrazine-preconditioned bone marrow mesenchymal stem cells promote microglia/macrophage M2 polarization and enhance neurogenesis in rats with ischemic stroke

**DOI:** 10.1186/s13287-025-04574-1

**Published:** 2025-08-26

**Authors:** Shihui Mao, Ting Lan, Yimei Sun, Lin Li, Weifeng Jiang, Jiadong Xu, Yan Feng, Huiqin Hu, Yan Fang, Lanxi Xu, Lisheng Chu

**Affiliations:** Department of Physiology, School of Basic Medical Sciences, The First Affiliated Hospital of Zhejiang Chinese Medical University (Zhejiang Provincial Hospital of Chinese Medicine), Zhejiang Chinese Medical University, Hangzhou, 310053 China

**Keywords:** Bone marrow mesenchymal stem cells, Extracellular vesicles, Tetramethylpyrazine, MiR-486, Microglia/macrophages, Neurogenesis, Ischemic stroke

## Abstract

**Background:**

Bone marrow mesenchymal stem cell-derived extracellular vesicles (BMSC-EVs) show therapeutic promise for ischemic stroke (IS). Preconditioning MSCs with drugs can modulate the cargo composition and function of their derived EVs. This study investigated the therapeutic effects and underlying mechanisms of EVs derived from tetramethylpyrazine (TMP)-preconditioned BMSCs (TMP-BMSC-EVs) in IS.

**Methods:**

EVs were isolated from BMSCs pretreated with or without TMP by differential centrifugation. The therapeutic efficacy of EVs was evaluated in a rat model of middle cerebral artery occlusion (MCAO) through neurological function assessments and infarct volume quantification. The expression of miR-486 and its roles in regulating microglia/macrophage polarization and neurogenesis, as well as the mechanistic targets, were examined by real-time quantitative polymerase chain reaction (RT-qPCR), immunofluorescence staining, and Western blotting.

**Results:**

TMP-BMSC-EVs exerted superior therapeutic efficacy compared to BMSC-EVs. Mechanistically, TMP-BMSC-EVs were enriched with miR-486, which promoted microglia/macrophage M2 polarization and neurogenesis, while downregulating phosphatase and tensin homolog (PTEN) and phosphorylated NF-κB (p-NF-κB) protein levels, and upregulating phosphorylated Akt (p-Akt) expression. Transfection with a miR-486 inhibitor abolished the beneficial effects of TMP-BMSC-EVs, which could be counteracted by the PTEN inhibitor bisperoxovanadium (bpV).

**Conclusions:**

TMP-BMSC-EVs could significantly promote neural repair by driving microglia/macrophage M2 polarization and enhancing neurogenesis through miR-486-mediated PTEN inhibition, thereby offering a promising treatment strategy for IS.

**Supplementary Information:**

The online version contains supplementary material available at 10.1186/s13287-025-04574-1.

## Graphical Abstract

The mechanism of TMP-BMSC-EVs promote function recovery in IS

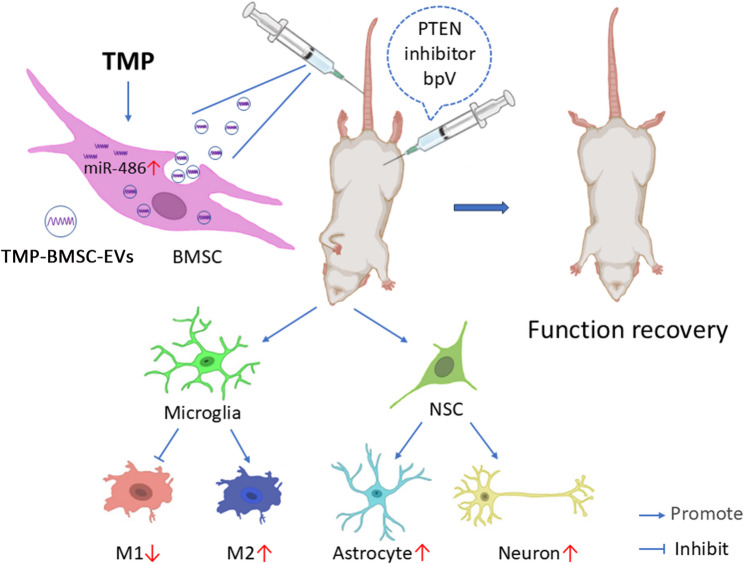

## Introduction

Stroke represents the most widespread disease affecting the central nervous system and stands as the primary underlying factor contributing to mortality and disability in the adult population, presenting a grave threat to human life and well-being [1, 2]. The categorization of stroke encompasses ischemic stroke (IS) and hemorrhagic stroke (HS), with IS constituting approximately 80% of all stroke cases [3]. Currently, intravenous thrombolysis and endovascular mechanical thrombolysis are effective methods for treating acute IS. However, there are restrictions due to the tight treatment schedule and hemorrhagic transformation, merely a small propotion of patients acquired positive outcomes from such treatments [4]. Therefore, there is an acute need for therapeutic approaches to repair ischemic brain injury.

Neural repair after cerebral ischemia is an intricate process comprising a sequence of events such as neuroinflammation, angiogenesis, neurogenesis, axon regeneration and synaptic plasticity [5]. Microglia is an immune cell that resides in the brain. Following cerebral ischemia, microglia and infiltrating macrophages are promptly activated and appear as inflammatory M1 and anti-inflammatory M2 [6]. Microglia/macrophages of the M1 phenotype are responsible for the secretion of a range of pro-inflammatory mediators that include tumor necrosis factor-α (TNF-α), Interleukin-1β (IL-1β), and IL-6, which are of great importance in the inflammatory response. In contrast, M2 microglia/macrophages release IL-10, transforming growth factor-β (TGF-β), brain-derived neurotrophic factor (BDNF), and vascular endothelial growth factor (VEGF), aiding inflammation resolution and brain tissue repair [7, 8]. Adult neural stem cells (NSCs), primarily situated within the subventricular zone (SVZ) and the subgranular zone (SGZ) of the dentate gyrus, are predominantly found in these specific regions [9, 10]. After focal cerebral ischemia, NSCs within the SVZ proliferate, migrate towards the area of ischemic damage, differentiate and develop into neurons, and establish synaptic connections with residual neurons, thereby facilitating neurorepair and functional recovery [11, 12]. Enhancing the M2 polarization, along with stimulating endogenous neurogenesis, contributes to neural regeneration and functional improvement in IS.

Stem cell-based therapy has great potential applications in the management of IS [13]. Mesenchymal stem cells (MSCs), present in a diverse range of tissues, like bone marrow (BM), umbilical cord, adipose tissue, are easily accessible and can rapidly proliferate, which make them excellent candidates for stem cell therapy [14]. Among them, the implantation of bone marrow-derived mesenchymal stem cells (BMSCs) has been established as a secure and efficacious strategy for the management of IS [15]. In the course of the last several decades, increasing evidence has suggested that BMSCs transplantation promoted neural repair after IS mainly by secreting neurotrophic factors and extracellular vesicles (EVs), rather than cell differentiation [16, 17]. Recent studies have found that EVs derived from BMSCs (BMSC-EVs) exhibit similar therapeutic effects to BMSCs in the treatment of IS, but with advantages such as low immunogenicity, good stability, readily permeable to the blood barrier, and low risk of tumorigenesis [18].

EVs are nanoparticles containing lipids, proteins, mRNA and microRNAs (miRs), which mediate intercellular communication [19, 20]. Accumulating evidence revealed that BMSC-EVs alleviated brain damage and promoted neural repair after IS mainly by transferring miR [14, 21]. Our studies have shown that BMSC-EVs delivering miR-21-5p or miR-486 promote angiogenesis and ameliorate brain damage after IS [22, 23]. Recent research has highlighted that preconditioning MSCs with hypoxia, chemical agents, or pharmacological treatments can modify the miR composition of MSC-EVs, thus boosting their therapeutic potential. For example, EVs from hypoxic-preconditioned MSCs alleviated cerebral ischemic injury through miR‑214‑3p/ phosphatase and tensin homolog (PTEN) signaling pathway [24]. Another study indicated that tanshinone IIA augmented the efficacy of exosomes derived from MSCs in treating myocardial ischemia-reperfusion injury through upregulating the abundance of exosomal microRNA-223-5p [25]. Tetramethylpyrazine (TMP) is the main active ingredient of traditional Chinese herb Rhizoma Chuanxiong (Chuanxiong), which is a commonly utilized treatment in clinical settings for cardiovascular and cerebrovascular disorders [26, 27]. Our earlier research demonstrated BMSCs pretreated with TMP enhanced angiogenesis and neurogenesis as a means to facilitate functional restoration in rats suffering from focal cerebral ischemia [28, 29]. However, the role of EVs derived from TMP-preconditioned BMSCs (TMP-BMSC-EVs) in neural repair after IS and their associated mechanisms needs further elucidation.

In this study, we hypothesized that TMP preconditioning enhanced the therapeutic efficacy of BMSC-EVs by enriching miR-486 within them, which drived microglia/macrophage M2 polarization and promoted neurogenesis via regulation of the PTEN/Akt signaling pathway. To test this hypothesis, we first explored the therapeutic effects of TMP-BMSC-EVs on IS, along with their influence on microglia/macrophage polarization and neurogenesis. We further investigated whether TMP-BMSC-EVs exerted their protective effects via miR-486-mediated targeted inhibition of PTEN. This study identifies a new mechanism by which TMP preconditioning enhances the neuroprotective effects of BMSC-EVs through miR-486/PTEN axis, providing a promising strategy for IS treatment.

## Methods

### Animals

Male Sprague-Dawley rats, weighing in the range of 290 ± 10 g (aged 6 to 8 weeks) and 90 ± 10 g (aged 4 weeks), were sourced from Shanghai SLAC Laboratory Animal Co., Ltd. They were placed in a regulated environment with supply of food and water ad libitum. The rats were housed in groups of 5 per cage under standard conditions, and cage locations were randomly assigned and rotated periodically. The temperature was held at 22 ± 2℃, with relative humidity of 40%-60%, and exposed to a 12-hour light-dark cycle. This research abides by the regulations set by the National Institutes of Health for the Care and Use of Laboratory Animals and received ethical clearance from the Experimental Animal Care and Ethics Committee of Zhejiang Chinese Medical University (Approval No. IACUC-20181126-13). The work has been reported in line with the ARRIVE guidelines 2.0.

### Cultivation of BMSCs

BM of the rats were harvested from the femurs and tibias and were rinsed by chilled Dulbecco’s Modified Eagle Medium/Nutrient Mixture F-12 (DMEM/F12, Gino Bio-Pharm Technology, Zhejiang, China). The harvested BM was then enzymatically dissociated into suspension of single cells, and cultivated in DMEM/F12 containing serum from fetal bovine (FBS), at a concentration of 10% (GEMINI, CA, USA). The culture medium was initially changed after 48 h and thereafter refreshed every 3-day. Once the cells reached 80%-90% confluence, they were subcultured at a 1:2 split ratio [30]. For experimental purposes, BMSCs from passages 3 to 5 were utilized to guarantee consistent phenotypic and functional properties.

### miR-486 inhibitor/nc transfection

The miR-486 inhibitor, together with a negative control (NC) sequence, was synthesized by RiboBio (Guangzhou, China), with the following: 5’-AGGACAUGACUCGACGGGGCUC-3’ for the inhibitor and 5’-AAACAGAUGUGUUUUCAUGAC-3’ for the NC. When BMSCs reached confluence of 80%, their transfection with 100 nM of either the miR-486 inhibitor or NC was carried out by Lipofectamine 2000 reagent and Opti-MEM medium (Invitrogen, CA, USA). 6 h post-transfection, the transfection medium was exchanged with DMEM/F12 medium that was supplemented with 10% EV-free FBS [31, 32]. Conditioned media from the transfected cells were harvested and centrifuged according to the previous description.

### TMP pretreatment

When the BMSCs reach 70%-80% confluence, discard the medium. Then, they were incubated in DMEM/F12 medium to which 10% EV-free serum and 50 µmol/L TMP (Aladdin, Shanghai, China) have been added at a temperature of 37℃ and under 5% CO_2_ conditions for 48 h [28].

### Extraction and characterization of EVs

The supernatants of BMSCs preconditioned or unpreconditioned with TMP were collected. Ultracentrifugation was used to isolate BMSC-EVs and TMP-BMSC-EVs (Thermo Fisher Scientific, MA, USA). The supernatants underwent sequential centrifugation: first at 300 ×g for 10 min, then 2000 ×g for 10 min, and lastly at 10,000 ×g for 30 min at 4℃, discarding the precipitates. Once the filtration through a 0.22 μm microporous membrane was completed, the resulting filtrate underwent high-speed centrifugation with force of 100,000 ×g for 2 h, maintained at 4℃. The pellet obtained was redispersed in 100 µL of phosphate-buffered saline (PBS), and the level of sum total protein present was determined employing the Micro BCA Protein Assay Kit (Thermo Fisher Scientific), prior to being stored at a temperature of minus 80℃.

To characterize EVs, various techniques were employed to analyze their shape, distribution of particle sizes, and those surface proteins like CD9, CD63, and ALIX. Particle structure was analyzed via transmission electron microscopy (TEM, Hitachi, Tokyo, Japan) and particle size distribution was measured using a Malvern Zetasizer Ultra (Malvern Instruments, Malvern, UK). Specific surface markers of EVs were identified through Western blot analysis [23, 29].

### Cellular internalization of EVs

Following the instructions supplied with the kit (MIDI67, Sigma-Aldrich, MA, USA), EVs were stained using the PKH67 dye [33]. The labeled EVs, at a density of 30 µg/mL, the samples were cultivated with microglia or NSCs for 12 h, followed by immobilization for 20 min using 4% paraformaldehyde. They were then subjected to a 15-minute treatment with 0.1% Triton X-100 to make them permeable and 5-minute staining with 4’,6-diamidino-2-phenylindole (DAPI) solution (Southern Biotech, Alabama, USA). The fluorescent labeling was then visualized and captured using an inverted fluorescence-based microscope (Leica DMIL, Wetzlar, Germany).

### Establishment of middle cerebral artery occlusion (MCAO) model

The MCAO model was created by following the procedure outlined by Longa et al. [34]. Isopentane anesthesia was used to render rats unconscious. Then, the right common carotid artery (CCA), external carotid artery (ECA), and internal carotid artery (ICA) underwent isolation and exposure. A 4 − 0 nylon suture was fed into the ICA via the ECA, advancing approximately 18–20 mm to obstruct blood circulation within the middle carotid artery (MCA). Following 90 min of occlusion, the suture was meticulously retracted to facilitate reperfusion. In the sham-operated group, all surgical procedures were carried out except for the insertion of the nylon suture. During the process, a 37℃ heating blanket was employed to keep the rats’ physiological stability.

### Experimental grouping and treatment

A total of 132 male SD rats (weighing 280–300 g) were used in this study. Dead individuals were excluded. Except the sham-operated group, the rats that underwent successful modeling were randomized grouped using the computer-based random number generator, comprising 12 rats per subgroup. Experimental grouping was performed as follows: Experiment 1: Sham-operated group, MCAO group, BMSC-EVs group, TMP-BMSC-EVs group. Experiment 2: Sham-operated group, MCAO group, BMSC-EVs group, TMP-BMSC-EVs group, TMP-inhibitor-EVs (TMP-BMSC-EVs transfected with miR-486 inhibitor) group, TMP-NC-EVs (TMP-BMSC-EVs transfected with miR-486 inhibitor NC) group, TMP-inhibitor-EVs + bisperoxovanadium (a PTEN inhibitor, bpV) group. EVs (100 µg/per rat) were dissolved in PBS (0.5 mL) and subsequently delivered through the vein in the tail, 24 h after the onset of reperfusion [23]. Rats in the sham-operated group and MCAO group were injected with PBS (0.5 mL) through the tail vein. PTEN inhibitor bpV (0.2 mg/kg, S865101, Selleck, TX, USA) [68] and 5-bromo-2’-deoxyuridine (BrdU, 50 mg/kg, 116M4799V, Sigma-Aldrich) were intraperitoneal injection once a day for 14 days [28]. The experimental rats were euthanized via cervical dislocation following anesthesia with isopentane to ensure they were unconscious.

### Neurological function evaluation

All experiments were performed under blinded conditions, ensuring that the researchers were unaware of the animal grouping, and assessed neurological outcomes at 1, 3, 7, and 14 days post-reperfusion. The modified neurological severity score (mNSS) serves as a scoring system used for evaluating functional recovery after neurological injury [35]. Scores from 0 to 18 represent normal to neurological deficits. Table 1 outlines the specific criteria for the mNSS. Rats with scores of 10–14 were considered successful in model induction. In addition, sensory-motor coordination is assessed with the corner test [36]. This consists of placing each rat in a V-shaped device consisting of two wooden boards at an angle of 30°. When the rat reaches the corner, the whiskers on both sides are stimulated and the body is lifted and turned over. Record the results of the number of left and right turns in 10 times per rat.

**Table 1:** The modified Neurological Severity Score (mNSS)

Items	score
Motor tests	
Raising rat by tail test	
Forelimb flexion	1
Hind limb flexion	1
Angle of head rotation > 10 degrees in 30 s	1
Placing the rat on the ground	
Normal movement	0
Cannot walk in a straight line	1
Turns in a circle in the direction of the hemiplegic side	2
Leaning towards hemiplegic side	3
Sensory tests	
Placement test (visual and tactile tests)	1
Proprioceptive test (deep sensation, pressing mouse paw towards the edge of the table to stimulate limb muscles)	1
Balance beam test	
Stabilising balance posture	0
Hold on to the side of the balance beam	1
Holding on to the balance beam with one limb slipping off the beam	2
Clutching the balance beam and sliding both limbs off the balance beam, or rotating on the balance beam (> 60 s)	3
Attempts to balance on balance beam but falls (> 40 s)	4
Attempts to balance on balance beam but falls (> 20 s)	5
Falling, no attempt to balance or hang on balance beam (< 20 s)	6
Loss of reflexes and paradoxical movements	
Auricular reflexes (head shaking on touching the ear canal)	1
Corneal reflex (blinking when touching the cornea with a cotton swab)	1
Startle reflex (motor response to noise of fast bouncing cardboard)	1
Seizures, myoclonus, dystonia	1

### Infarct volume detection

Anesthesia was administered to surviving rats 14 days after cerebral ischemia. Precooled 0.9% sodium chloride solution and 4% polyformaldehyde were perfused via the left ventricle. Overnight, the brains, once removed, were positioned in a 4% paraformaldehyde solution. Subsequently, they underwent dehydration processes involving 20% and 30% sucrose solutions. With a cryogenic microtome (MH525 NX, Thermo Fisher Scientific), frozen sections were cut, measuring 10 and 20 μm in thickness. After being stained for 7 min with a solution containing 1% toluidine blue (T3260, Sigma-Aldrich), the 20 μm sections were then dehydrated in 75%, 95%, and 100% alcohol, each for 2 min in succession, to measure infarct volume. Calculation of each section’s infarct area was done employing Image-pro Plus 6.0. The ratio of volume of infarction was computed using the formula: [volume of left hemisphere - (volume of.

right hemisphere - right infarct volume)] / volume of left hemisphere × 100%.

### Double immunofluorescence staining

For immunofluorescence analysis, sections of the brain measuring 10 μm in thickness were utilized. When performing double staining for CD16/32/Iba1 and CD206/Iba1, the sections underwent treatment with 0.3% Triton X-100 for 30 min. Subsequently, they were incubated at a temperature of 4℃ overnight with the primary antibodies, which comprised goat anti-CD206 (AF-2535, 1:100, R&D Systems, MN, USA), mouse anti-CD16/32 (553141, 1:200, BD Pharmingen, CA, USA), and rabbit anti-Iba1 (019-19741, 1:500, Wako, Japan). Subsequent day, the tissue sections were subjected to 1 h incubation period at 37℃ with the corresponding secondary antibodies: fluorescein isothiocyanate (FITC)-labeled goat anti-mouse IgG (115001003), donkey anti-goat IgG (705001003), and Cy3-labeled donkey anti-rabbit IgG (711165152, 1:100, Jackson ImmunoResearch, USA).

Sections were incubated with 2 mol/L HCl for 30 min at 37℃ and then double-stained for BrdU/DCX, BrdU/NeuN and BrdU/GFAP. Neutralization was achieved using 0.1 mol/L boric acid (pH 8.5). Primary antibodies included sheep anti-BrdU (ab1893, 1:200, Abcam, MA, USA), goat anti-doublecortin (DCX, sc-8066, 1:200, Santa Cruz, CA, USA), rabbit anti-NeuN (MABN140, 1:500, Millipore, MA, USA), and mouse anti-GFAP (sc-51908, 1:100, Santa Cruz). Overnight incubation at 4℃, and sections were dealt with Cy3-labeled donkey anti-sheep IgG (713165147), FITC-labeled goat anti-mouse IgG (115001003), donkey anti-goat IgG (705001003), and goat anti-rabbit IgG (111095003, Jackson ImmunoResearch, PA, USA) about 1 h at 37 °C [37]. Fluorescent images were acquired via a microscope equipped for fluorescence observation (DMIL, Leica Microsystems, Germany). Positive cells in three randomly selected regions per sample were counted for subsequent analysis.

### Real-time quantitative polymerase chain reaction (RT-qPCR)

Total RNA was reverse transcribed into cDNA according to the instructions provided by the Mir-X™ miRNA First-Strand Synthesis Kit (638315, TaKaRa, Tokyo, Japan) and PrimeScript RT Master Mix (RR036A, TaKaRa). Quantitative PCR (qPCR) was conducted using the TB Green Premix Ex Taq Kit on a CFX96 Real-Time System (Bio-Rad, CA, USA) [37]. Relative gene expression levels were calculated by the 2^−ΔΔCT^ method, with GAPDH or U6 serving as internal references. Primer sequences, synthesized by Sango (Shanghai, China), are provided in Table 2.

**Table 2:** The primers sequences used for qPCR

Genes	Forward primer sequence (5’-3’)	Reverse primer sequence (5’-3’)
iNOS	ATGGCTCCTTCAAAGAGGCA	CTATTTCCTTTGTTACGGCTTCCA
IL-6	AAATCTGCTCTGGTCTTC	AGGGTTTCAGTATTGCTC
IL-1β	AATGCCTCGTGCTGTCTGA	GGATTTTGTCGTTGCTTGTCTC
Arg-1	TCCTTAGAGATTATCGGAGCG	GTCTTTGGCAGATATGCAGG
IL-10	CAAGGCAGTGGAGCAGGTGA	CCGGGTGGTTCAATTTTTCATT
TGF-β	CTGAGTGGCTGTCTTTTGACGTC	AAGCCCTGTATTCCGTCTCCTTG
GAPDH	GCCAAGGCTGTGGGCAAGGT	TCTCCAGGCGGCACGTCAGA
rno-miR-486	CGCGTGAGAACTGAATTCCATGGGTT	
U6	CCGAGAGAAGATTAGCATGGCCCCTG	

### Western blotting

Brain tissue was lysed using RIPA lysis buffer. Protein concentrations were quantified using a BCA protein assay kit. Each sample was subjected to separation on a 10% SDS-PAGE gel (Beyotime, Shanghai, China) and subsequently transferred onto a PVDF membrane. The membranes were incubated with primary antibodies, and gently shaken overnight at 4℃, including rabbit anti-CD9 (13174, 1:1000, Cell Signaling Technology, MA, USA), anti-CD63 (BS72936, 1:1000, Bioworlde, MN, USA), anti-ALIX (ab275377, 1:1000, Abcam), anti-PTEN (9559, 1:1000, Cell Signaling Technology, MA, USA), anti-p-Akt (4060, 1:1000, Cell Signaling Technology, MA, USA), anti-Akt (9272, 1:1000, Cell Signaling Technology, MA, USA), anti-NF-κB p65 (8242, 1:1000, Cell Signaling Technology, MA, USA), anti-p-NF-κB p65 (3033, 1:1000, Cell Signaling Technology, MA, USA), mouse anti-glyceraldehyde 3-phosphate dehydrogenase (GAPDH) (EM1101, 1:1000, Huabio, Zhejiang, China), and anti-β-actin (sc-47778, 1:1000, Santa Cruz). The following day, membranes were incubated with appropriate secondary antibodies: goat anti-rabbit IgG (BK-R050, 1:2000, Bioker Biotechnology, Zhejiang, China) and goat anti-mouse IgG (BK-M050, 1:2000, Bioker Biotechnology, Zhejiang, China) [38]. Protein bands were visualized using a gel imaging system (Bio-Rad), and the grey values of the bands were quantified using ImageJ software (NIH, MD, USA).

### Enzyme-linked immunosorbent assay (ELISA)

Following rapid collection, brain tissues were immediately immersed in ice-cold PBS. The peri-infarct region was meticulously dissected on a pre-cooled platform (-20℃) and accurately weighed. Tissue homogenization was performed in ice-cold PBS at a 1:9 (w/v) ratio using a mechanical homogenizer. Subsequent centrifugation was performed at 2,000 ×g for 20 min at 4℃ to isolate the supernatant. The content of PTEN was measured using a commercial ELISA kit (MM-70708R2, Meimian, Jiangsu, China) according to the manufacturer’s protocol [39].

### Statistical examination

The data processed by SPSS 26.0 software (SPSS, IL, USA) were expressed as the mean ± standard deviation. We conducted the Shapiro-Wilk test to assess data normality. For datasets with a normal distribution, a one-way analysis of variance (ANOVA) was conducted, and subsequently, Tukey’s post hoc test was performed to evaluate inter-group differences. In cases where data were not normally distributed, the Kruskal–Wallis H test was carried out. *P* < 0.05 was regarded as statistically significant.

## Results

### Characterization and internalization of EVs

**Fig. 1:**
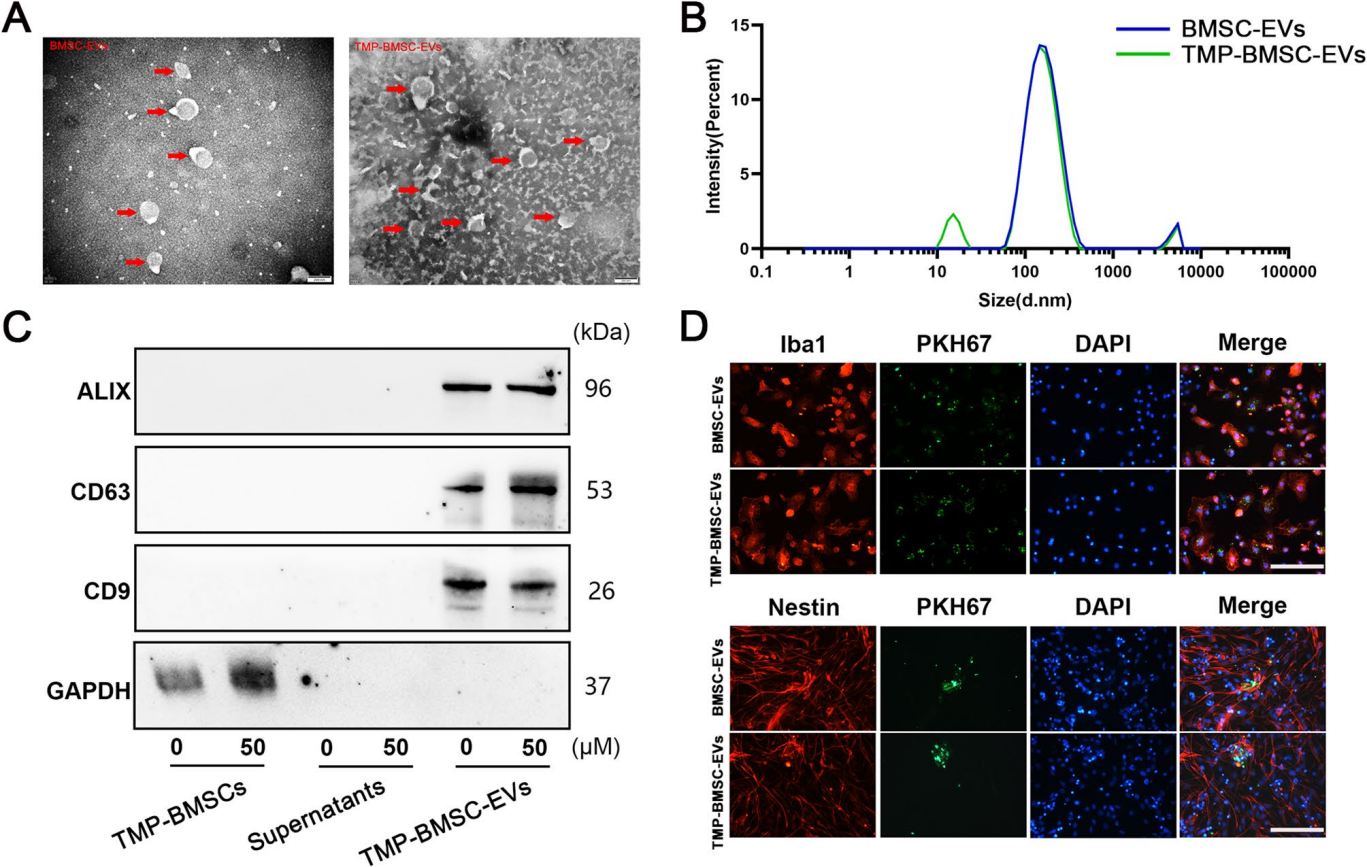
Characterization and cellular uptake of BMSC-EVs and TMP-BMSC-EVs. (**A**) Electron microscopy observation of BMSC-EVs and TMP-BMSC-EVs morphology. Scale bar = 200 nm. (**B**) Size distribution profiles of BMSC-EVs and TMP-BMSC-EVs, measured by dynamic light scattering. Data are presented as intensity (%) versus hydrodynamic diameter (d.nm). (**C**) Expression of EVs’ markers (Alix, CD63, CD9) and absence of GAPDH. (**D**) Representative immunofluorescence images showed that PKH67 labeled BMSC-EVs and TMP-BMSC-EVs can be uptaken by microglia and NSCs. Scale bar = 20 μm

The features of BMSCs and BMSCs-EVs have been described in our former work [23, 28]. In our investigation, we conducted a comparative analysis of the characteristics between BMSCs-EVs and TMP-BMSCs-EVs. TEM analysis revealed that both types of EVs exhibited shape that is either round or oval, encapsulated within a distinct double-layer membrane structure (Fig. 1A). Analysis conducted with Malvern instruments showed that the average diameters of BMSCs-EVs and TMP-BMSCs-EVs were 115.6–119.8 nm and 113.1–124.1 nm, respectively, with no significant changes observed in size distribution due to TMP preconditioning (Fig. 1B). Western blot analysis demonstrated that both types of EVs showed CD9, CD63, and ALIX, yet without GAPDH expression (Fig. 1C). The results verify that the vesicles, isolated through ultracentrifugation, are indeed EVs, appropriate for further experimentation.

To examine the cellular uptake of EVs, we co-cultivated the PKH67-marked EVs with microglia or NSCs for 12 h (Fig. 1D). The results confirmed that TMP-BMSCs-EVs along with BMSCs-EVs were effectively internalized by these two cell types.

### TMP-BMSC-EVs enhanced neurological function recovery and decreased infarct volume after IS

**Fig. 2:**
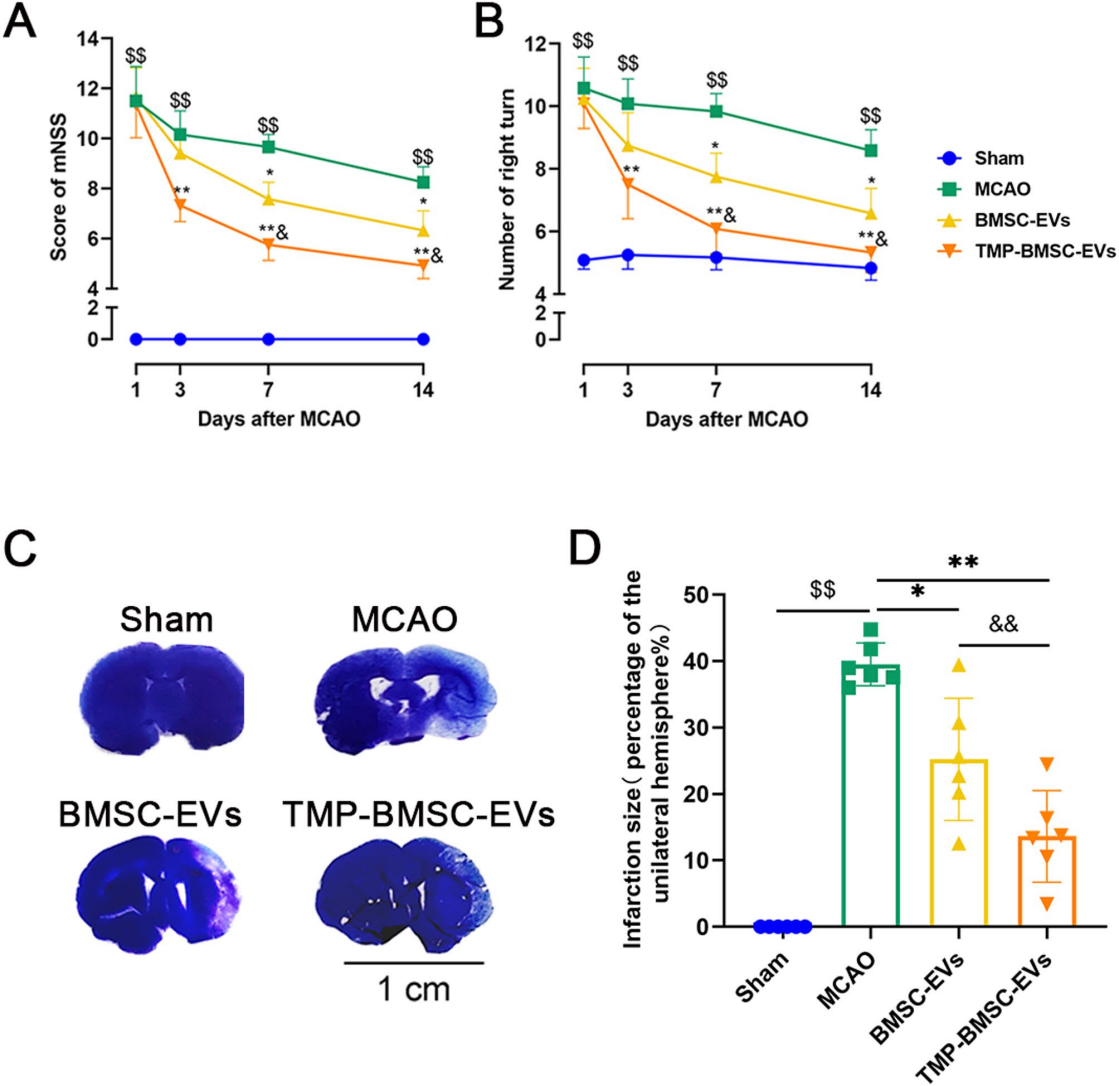
TMP-BMSC-EVs improved neurological function after IS in rats. (**A-B**) mNSS and the corner test. *n* = 12. (**C**) Representative images of brain slices by toluidine blue staining, Scale bar = 1 cm. (**D**) Quantification of infarct volume at 14th after MCAO. *n* = 6. ^$$^*p* < 0.01 vs. Sham group, ^*^p < 0.05 and ^**^p < 0.01 vs. MCAO group, ^&^*p* < 0.05 and ^&&^*p* < 0.01 vs. BMSC-EVs group

A cerebral ischemia/reperfusion injury rat model was set up to study TMP-BMSC-EVs’ neuroprotective effects. 24 h after reperfusion, rats received tail vein injections of either BMSC-EVs or TMP-BMSC-EVs. Neurological function was assessed on the 1st day, 3rd day, 7th day, and 14th day after ischemia using the mNSS and the corner test. No expected or unexpected adverse events were observed during the experiment. Results suggested that both BMSC-EVs and TMP-BMSC-EVs notably reduced mNSS scores and minimized the number of right turns in ischemic rats from day 3 onward (^*^p < 0.05, ^**^p < 0.01). However, TMP-BMSC-EVs demonstrated a more pronounced effect starting on day 7 (Fig. 2A-B, ^&^*p* < 0.05). Assessment of cerebral infarct volume by toluidine blue staining. Compared to the untreated model, both EVs treatments significantly reduced infarction volume (^*^p < 0.05, ^**^p < 0.01), with TMP-BMSC-EVs showing superior efficacy (Fig. 2C-D, ^&&^*p* < 0.01). These findings suggest that TMP-BMSC-EVs provide enhanced neuroprotection in IS compared to BMSC-EVs.

### TMP-BMSC-EVs inhibited microglia/macrophages polarization towards M1 and enhanced microglia/macrophages polarized to M2 after IS

**Fig. 3:**
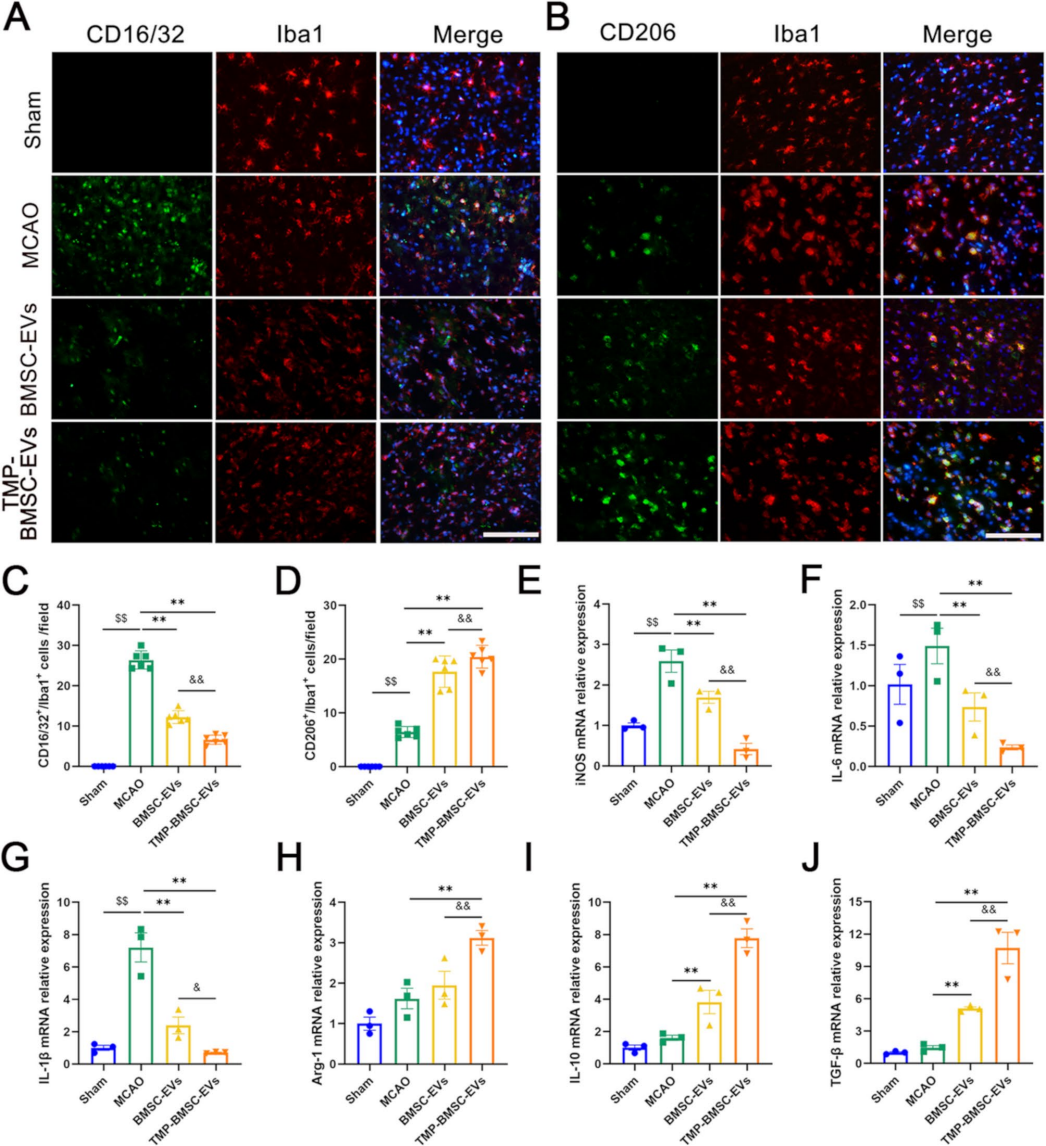
TMP-BMSC-EVs promoted microglia/macrophages phenotype transition from M1 to M2 after MCAO in rats. (**A-B**) Representative images of CD16/32/Iba1, CD206/Iba1 double immunofluorescence staining in the peri-infarct regions after MCAO in rats. Scale bar = 100 μm. *n* = 6. (**C-D**) Quantification of the number of CD16/32^+^/Iba1^+^ and CD206^+^/Iba1^+^ cells. (**E-J**) The mRNA expression of M1 markers (iNOS, IL-1β, and IL-6) and M2 markers (Arg-1, IL-10, and TGF-β) were measured by RT-qPCR. *n* = 3. ^$$^*p* < 0.01 vs. Sham group, ^**^p < 0.01 vs. MCAO group, ^&^*p* < 0.05 and ^&&^*p* < 0.01 vs. BMSC-EVs group

To explore the impact of TMP-BMSC-EVs on M1/M2 microglia/macrophages polarization, biomarker expression was analyzed using immunofluorescence staining and RT-qPCR at 14 days post-ischemia (Fig. 3A-D). Immunofluorescence staining demonstrated that treatment with both BMSC-EVs and TMP-BMSC-EVs led to a substantial reduction with the quantity of CD16/32^+^/Iba1^+^ cells, indicative of M1 microglia/macrophages, while concurrently increasing population of CD206^+^/Iba1^+^ cells, representative of M2 type of microglia/macrophages (^**^p < 0.01). Correspondingly, RT-qPCR analysis indicated that BMSC-EVs and TMP-BMSC-EVs significantly downregulated the mRNA levels of M1-associated markers, which included iNOS, IL-1β, and IL-6. At the same time, they notably raised the mRNA expression of markers that are associated with M2, such as Arg-1, IL-10, and TGF-β (Fig. 3E-J, ^**^p < 0.01). Notably, the therapeutic effects observed with TMP-BMSC-EVs were significantly superior to those achieved with BMSC-EVs (^&^*p* < 0.05, ^&&^*p* < 0.01), underscoring their enhanced efficacy.

### TMP-BMSC-EVs enhanced neurogenesis in IS rats

**Fig. 4:**
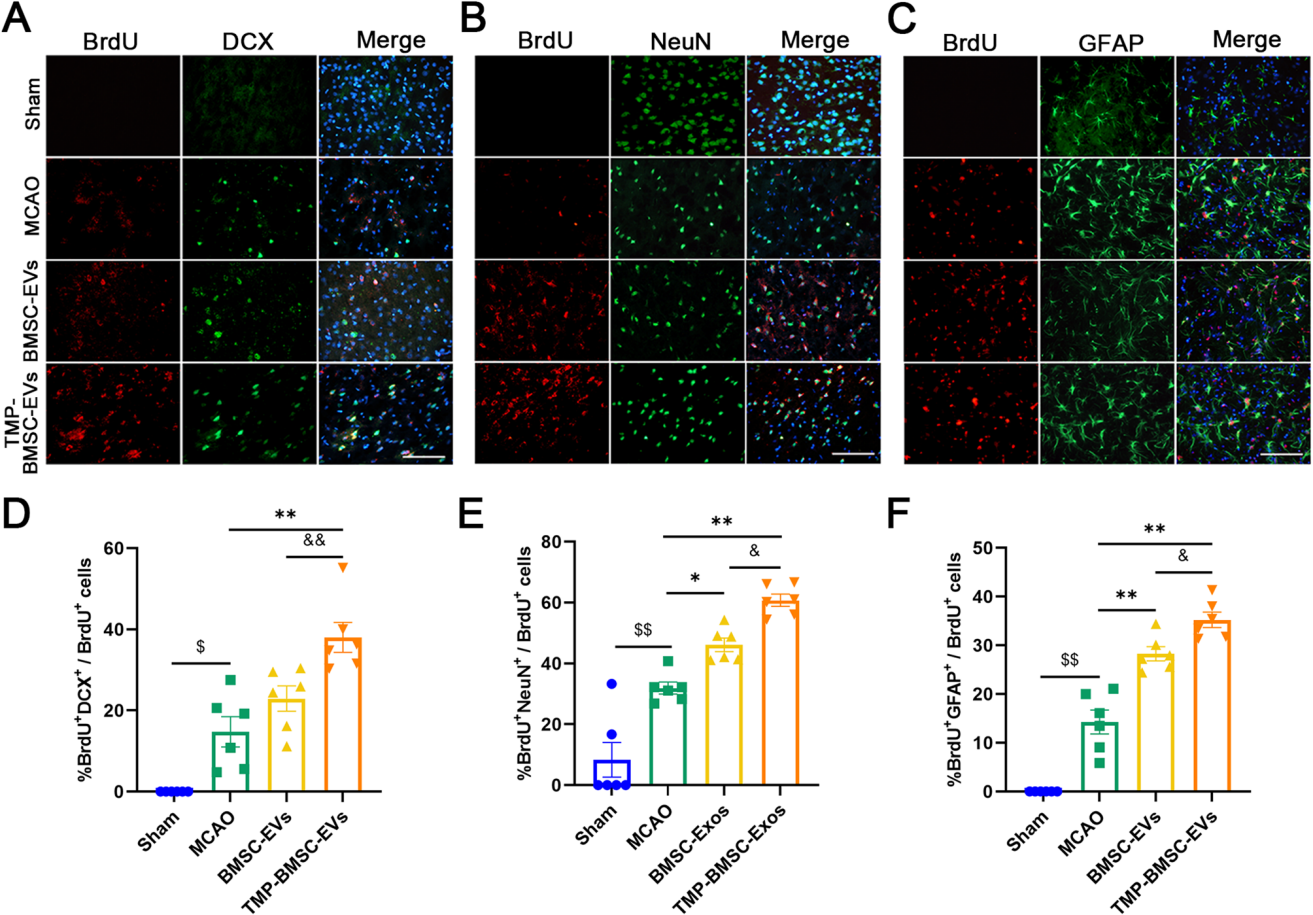
TMP-BMSC-EVs exhibited better effects in promoting neurogenesis after MCAO in rats. (**A-C**) Representative images of BrdU/DCX, BrdU/NeuN, and BrdU/GFAP double immunofluorescence staining in the peri-infarct regions after MCAO. (**D-F**) Proportions of BrdU^+^/DCX^+^, BrdU^+^/NeuN^+^, and BrdU^+^/GFAP^+^ within total BrdU^+^ cells. *n* = 6. ^$$^*p* < 0.01 vs. Sham group, ^*^p < 0.05 and ^**^p < 0.01 vs. MCAO group, ^&^*p* < 0.05 and ^&&^*p* < 0.01 vs. BMSC-EVs group

Neurogenesis was evaluated by double-labeling immunofluorescence with BrdU combined with DCX, NeuN, and GFAP at 14 days subsequent to ischemia. The findings suggested that BMSC-EVs and TMP-BMSC-EVs can increase the count of BrdU^+^/DCX^+^, BrdU^+^/NeuN^+^ and BrdU^+^/GFAP^+^ cells (Fig. 4, ^*^p < 0.05, ^**^p < 0.01). Additionally, TMP-BMSC-EVs were superior to BMSC-EVs in promoting neurogenesis (^&^*p* < 0.05, ^&&^*p* < 0.01).

### TMP-BMSC-EVs regulated miR-486, PTEN, p-Akt, and p-NF-κB expression in IS rats

**Fig. 5:**
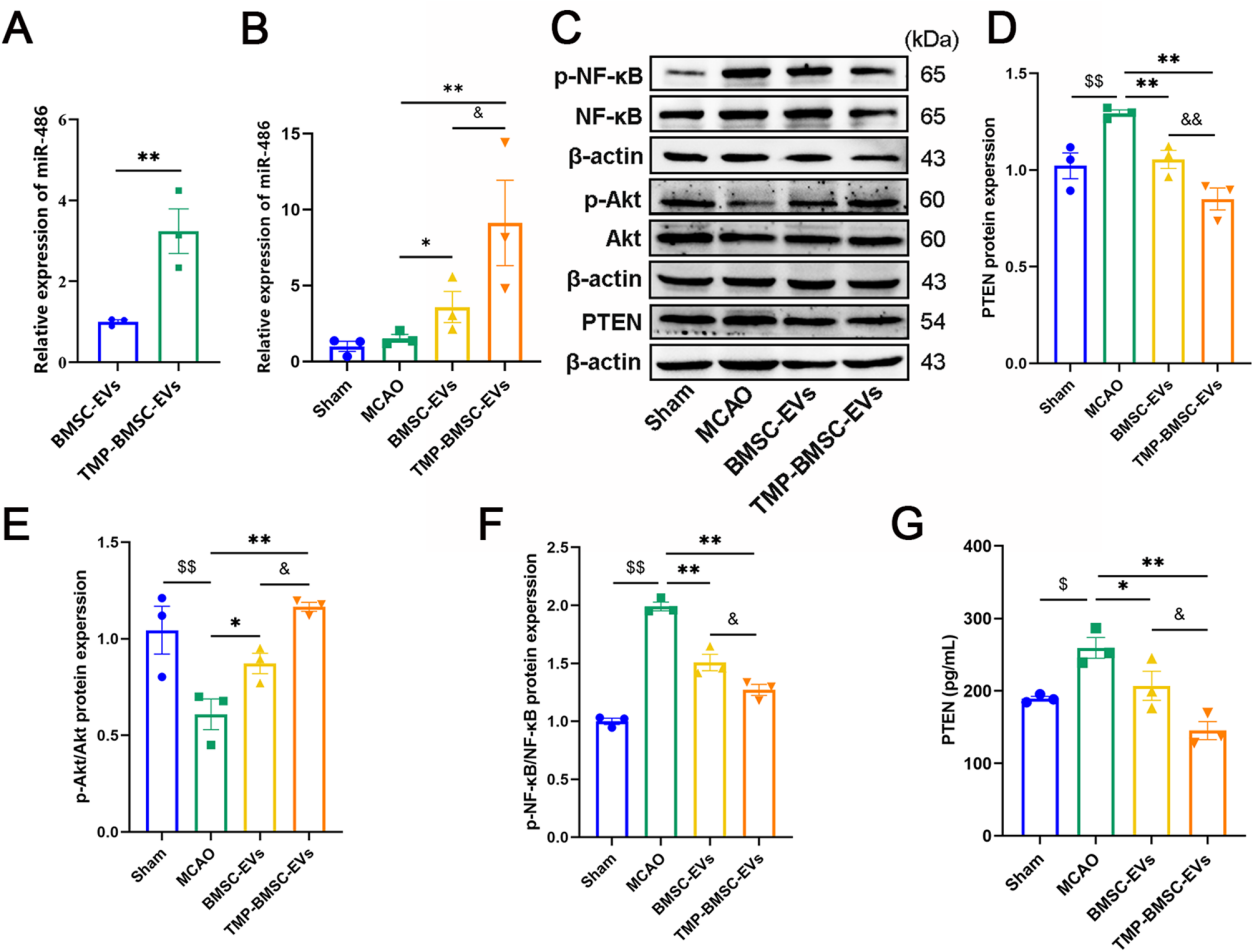
Effects of TMP-BMSC-EVs on miR-486 expression and PTEN/Akt signaling in IS rats. (**A**) The expression levels of miR-486 in BMSC-EVs pretreated with TMP (TMP-BMSCs). *n* = 3. ^**^p < 0.01 vs. BMSC-EVs group. (**B**) The expression of miR-486 was determined by RT-qPCR. (**C**) Representative bands of PTEN, p-Akt, Akt, p-NF-κB and NF-κB expressions by western blot. *n* = 3. (**D-F**) Semiquantitative analysis of PTEN, p-Akt, Akt, p-NF-κB and NF-κB. *n* = 3. (**G**) The content of PTEN in brain tissue of each group was determined by ELISA. *n* = 3. ^$^*p* < 0.05 and ^$$^*p* < 0.01 vs. Sham group, ^*^p < 0.05 and ^**^p < 0.01 vs. MCAO group, ^&^*p* < 0.05 and ^&&^*p* < 0.01 vs. BMSC-EVs group

Recent our lab findings indicate BMSC-EVs enhance angiogenesis and neurological recovery post-cerebral ischemia by suppressing PTEN through miR-486 [23]. However, whether TMP pretreatment enhances recovery from cerebral ischemic injury by elevating EV enriched miR-486 levels remain to be clarified. To address this, we initially analyzed the expression of miR-486 in both BMSC-EVs and TMP-BMSC-EVs. We conclude that TMP pretreatment significantly increased miR-486 content in BMSC-EVs (Fig. 5A, ^**^p < 0.01). Then, the expression of miR-486, PTEN, p-Akt, Akt, p-NF-κB, and NF-κB were detected in the peri-infarct region. The findings revealed that TMP-BMSC-EVs treatment markedly enhanced the expression of miR-486, p-Akt, while downregulating the protein levels of PTEN and p-NF-κB (Fig. 5B-F, ^&^*p* < 0.05, ^&&^*p* < 0.01). We also examined the content of PTEN by ELISA (Fig. 5G), which was consistent with the increase in its protein expression level. The findings suggest that TMP-BMSC-EVs may alleviate IS injury by upregulating EV enriched miR-486, thereby modulating the miR-486/PTEN/Akt signaling pathway.

### TMP-BMSC-EVs improved IS injury through miR-486/PTEN pathway

**Fig. 6:**
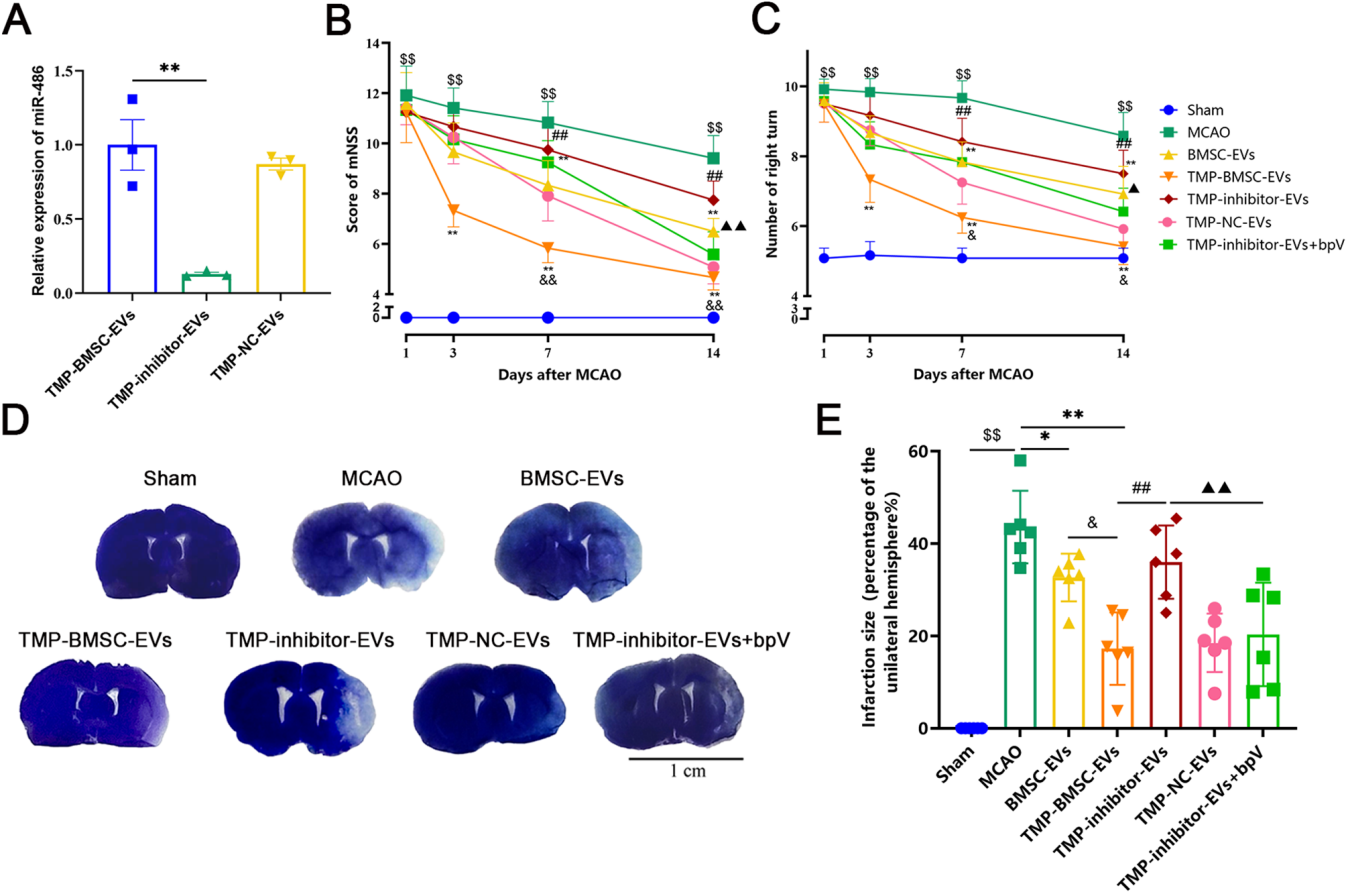
TMP-BMSC-EVs ameliorated cerebral ischemic injury in rats through miR-486/PTEN pathway. (**A**) The expression levels of miR-486 in TMP-BMSCs-EVs after miR-486 inhibitor transfection. *n* = 3. ^**^p < 0.01 vs. TMP-BMSC-EVs group. (**B-C**) mNSS and the corner test. *n* = 12. (**D**) Representative images of brain slices by toluidine blue staining. (**E**) Quantification of infarct volume at 14th after MCAO. *n* = 6. ^$$^*p* < 0.01 vs. Sham group, ^*^p < 0.05 and ^**^p < 0.01 vs. MCAO group, ^&^*p* < 0.05 and ^&&^*p* < 0.01 vs. BMSC-EVs group, ^##^*p* < 0.01 vs. TMP-BMSC-EVs group, ▲*p* < 0.05 and ▲▲*p* < 0.01 vs. TMP-inhibitor EVs group

To further explore the involvement of the miR-486/PTEN pathway in the therapeutic benefits of TMP-BMSC-EVs, EVs were isolated from TMP-preconditioned BMSCs transfected with either a miR-486 inhibitor (TMP-inhibitor-EVs) or a negative control (TMP-NC-EVs). RT-qPCR revealed lower miR-486 expression in TMP-inhibitor-EVs compared to TMP-BMSC-EVs (Fig. 6A, ^**^p < 0.01). Subsequently, rats were treated with these EVs via tail vein injection and concurrently administered the PTEN inhibitor bpV intraperitoneally. The neuroprotective effects of TMP-BMSC-EVs were validated through behavioral assessments and infarct volume measurements, which showed that these benefits were abolished when miR-486 was inhibited (Fig. 6B-E, ^##^*p* < 0.01). The inhibitory effects of TMP-inhibitor-EVs were counteracted by the PTEN inhibitor bpV (▲*p* < 0.05, ▲▲*p* < 0.01). These discoveries imply that TMP-BMSC-EVs lessen the effects of IS injury by targeting the miR-486/PTEN signaling pathway.

### TMP-BMSC-EVs switched M1-to-M2 microglia/macrophages phenotype and augmented neurogenesis after IS through miR-486/PTEN pathway

**Fig. 7:**
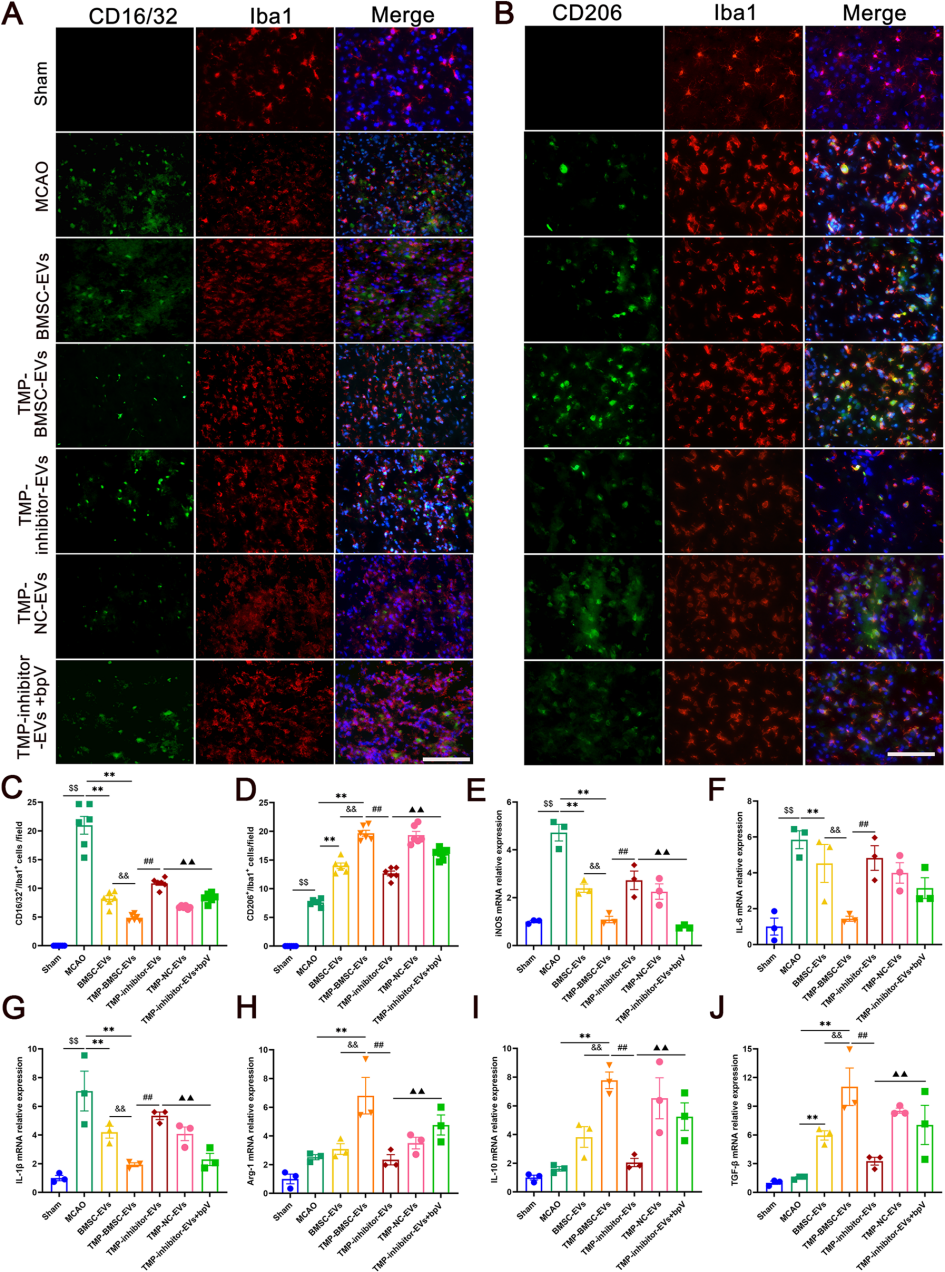
TMP-BMSC-EVs inhibited M1 polarization and promoted M2 polarization of microglia/macrophages through the miR-486/PTEN pathway. (**A-B**) Representative images of CD16/32/Iba1 (M1 marker) and CD206/Iba1 (M2 marker) double immunofluorescence staining in the peri-infarct regions in rats. Scale bar = 100 μm. *n* = 6. (**C-D**) Quantification of CD16/32^+^/Iba1^+^ and CD206^+^/Iba1^+^ cells. (**E-J**) The mRNA expression levels of iNOS, IL-6, IL-1β, Arg-1, IL-10, and TGF-β were determined by RT-qPCR. *n* = 3. ^$$^*p* < 0.01 vs. Sham group, ^**^p < 0.01 vs. MCAO group, ^&&^*p* < 0.01 vs. BMSC-EVs group, ^##^*p* < 0.01 vs. TMP-BMSC-EVs group, ▲▲*p* < 0.01 vs. TMP-inhibitor EVs group

**Fig. 8:**
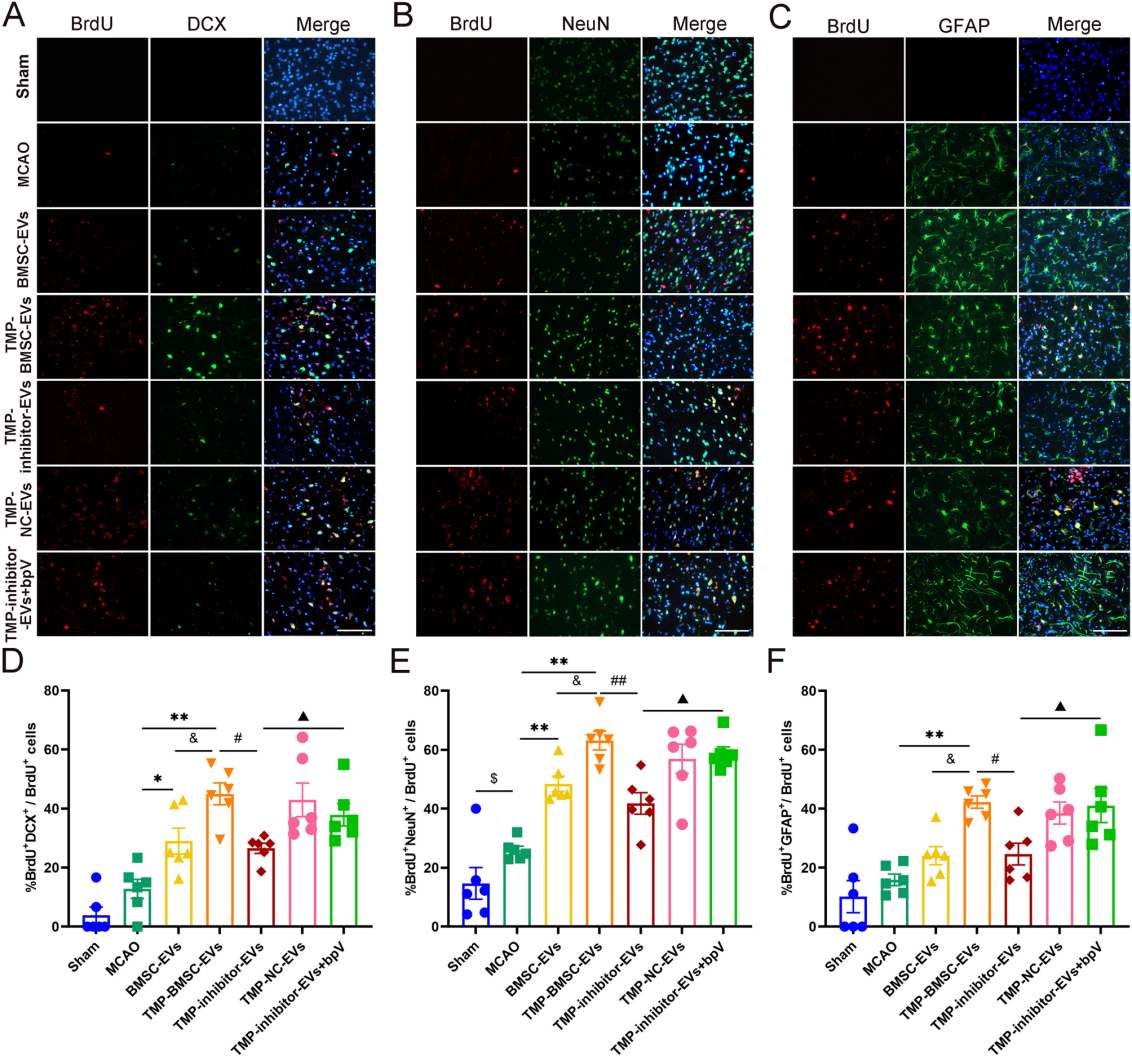
TMP-BMSC-EVs promoted neurogenesis through miR-486/PTEN pathway. (**A-C**) Representative images of BrdU/DCX, BrdU/NeuN, BrdU/GFAP double immunofluorescence staining. *n* = 6. (**D-F**) Proportions of BrdU^+^/DCX^+^, BrdU^+^/NeuN^+^, and BrdU^+^/GFAP^+^ within total BrdU^+^ cells. ^$^*p* < 0.05 vs. Sham group, ^*^p < 0.05 and ^**^p < 0.01 vs. MCAO group, ^&^*p* < 0.05 vs. BMSC-EVs group, ^#^*p* < 0.05 and ^##^*p* < 0.01 vs. TMP-BMSC-EVs group, ▲*p* < 0.05 vs. TMP-inhibitor EVs group

**Fig. 9:**
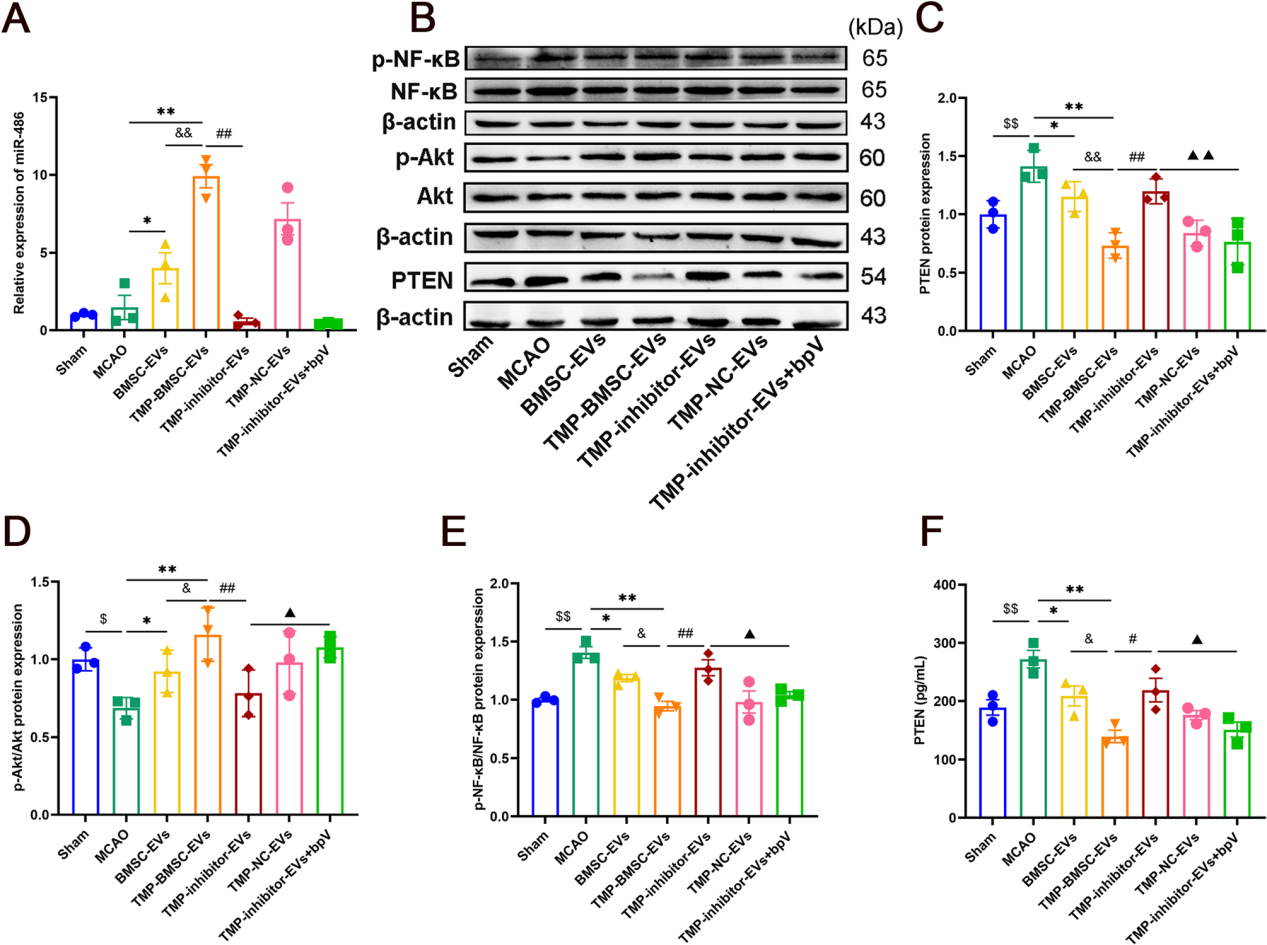
Effect of TMP-BMSC-EVs on PTEN, p-Akt, and p-NF-κB protein expression in the peri-infarct zone of cerebral ischemic rats by transporting miR-486. (**A**) The expression of miR-486 was determined by RT-qPCR. *n* = 3. (**B-E**) Protein levels of PTEN, p-Akt, Akt, p-NF-κB and NF-κB were assessed by western blot analysis. *n* = 3. (**F**) The content of PTEN in brain tissue of each group was determined by ELISA. *n* = 3. ^$^*p* < 0.05 and ^$$^*p* < 0.01 vs. Sham group, ^*^p < 0.05 and ^**^p < 0.01 vs. MCAO group, ^&^*p* < 0.05 and ^&&^*p* < 0.01 vs. BMSC-EVs group, ^#^*p* < 0.05 and ^##^*p* < 0.01 vs. TMP-BMSC-EVs group, ▲*p* < 0.05 and ▲▲*p* < 0.01 vs. TMP-inhibitor EVs group

As shown in Figs. 7 and 8, similar to previous results, TMP-BMSC-EVs promoted phenotypic transformation of microglia/macrophages from M1 to M2 and amplified neurogenesis after IS (^*^p < 0.05, ^**^p < 0.01), while miR-486 inhibitor blocked the effects of TMP-BMSC-EVs (^#^*p* < 0.05, ^##^*p* < 0.01), but PTEN inhibitor bpV reversed the effects of TMP-inhibitor-EVs (▲*p* < 0.05, ▲▲*p* < 0.01). Then we detected the miR-486, PTEN, p-Akt, Akt, p-NF-κB, and NF-κB expression in the peri-infarction area. Compared to TMP-BMSC-EVs, TMP-inhibitor-EVs markedly suppressed miR-486 expression, elevated PTEN and p-NF-κB protein levels, and reduced p-Akt expression (^##^*p* < 0.01). ELISA results showed that TMP-inhibitor-EVs significantly increased the content of PTEN (^#^*p* < 0.05). However, bpV counteracted these effects of TMP-BMSC-EVs (Fig. 9, ▲*p* < 0.05, ▲▲*p* < 0.01). The data indicate that TMP-BMSC-EVs facilitated the M1 to the M2 phenotype transition of microglia/macrophages and stimulated neurogenesis following IS via the miR-486/PTEN pathway.

## Discussion

Numerous studies have revealed that BMSC-EVs could alleviate cerebral ischemic injury and enhance nerve regeneration after IS [40–43]. Our previous research indicated that TMP pretreatment promoted the migration of transplanted BMSCs to the peri-ischemic area, increased angiogenesis, and improved neurological function recovery [28, 29]. Building on this foundation, the current study demonstrates that TMP-BMSC-EVs significantly enhanced neurological recovery, facilitated M1-to-M2 phenotypic transition of microglia/macrophages, and augmented neurogenesis in rats after IS. Crucially, mechanistic investigations revealed that these benefits were primarily mediated through the delivery of EV-enriched miR-486, which targeted and suppressed PTEN expression.

Similar to stem cell transplantation, EVs possess therapeutic effects and boast minimal immunogenicity, stability, and a capacity to effortlessly traverse the blood-brain barrier. Consequently, BMSC-EVs are recognized as promising candidates for IS repair therapy. However, the therapeutic efficacy of BMSC-EVs remains limited. Accumulating evidence has shown that the cellular microenvironment can alter the cargo in EVs, such as hypoxia, chemical reagents, and drug stimulation, to enhance their functions [44–46]. Compared with hypoxia and cytokine stimulation, pharmacological preconditioning offers distinct advantages for clinical translation due to its precise dosing and standardized protocols. For example, EVs derived from MSCs pretreatment with nicorandil promoted cardiac recovery from myocardial infarction [47]. while atorvastatin-preconditioned MSC-EVs accelerated wound healing in diabetic rats [48]. Similarly, active compounds from traditional Chinese medicine, such as tanshinone IIA [25]. and baicalin [49], have been shown to boost the therapeutic efficacy of MSC-EVs in other disease models. In this study, we demonstrated that preconditioning with TMP, a clinically utilized neuroprotective agent for cerebrovascular disorders [50, 51], significantly enhanced the therapeutic potential of BMSC-EVs in IS, as evidenced by superior neurological recovery and greater reduction in infarct volume compared to BMSC-EVs.

Neural repair following cerebral ischemia involves a highly intricate process that includes neuroinflammation, angiogenesis, and neurogenesis. Ample research supports the part played by M2-polarized microglia/macrophages in resolving inflammation and boosting brain tissue regeneration after central nervous system injury [52, 53]. Nonetheless, prior studies indicated that while microglia/macrophages initially polarize toward the anti-inflammatory M2 during the early IS, they progressively shift to the pro-inflammatory M1 in peri-infarct region [54, 55]. Endogenous neurogenesis triggered by IS facilitates brain repair and recovery of neurological functions [16]. In addition, more and more evidence suggests that M2 microglia/macrophages enhance neurogenesis by releasing neurotrophic factors, whereas M1 cells hinder this process by secreting pro-inflammatory cytokines [56]. Therefore, promoting microglial/macrophage M2 polarization and enhancing neurogenesis contribute to neural repairing and function rehabilitation after IS. Numerous studies have reported that BMSC-EVs could facilitate M2 polarization of microglia/macrophages, angiogenesis, neurogenesis, and neuroplasticity [57–59]. In this study, our results confirmed that BMSC-EVs facilitated M1-to-M2 transition and augmented neurogenesis post-ischemia. Importantly, TMP-BMSC-EVs exhibited significantly superior efficacy in both promoting M2 polarization and enhancing neurogenesis compared to BMSC-EVs.

EVs act as mediators of intercellular communication by delivering their encapsulated proteins, lipids, and miRNAs to recipient cells, thereby modulating the functions of those cells. Among the diverse cargos in EVs, miRs have attracted significant attention for their ability to either degrade target gene mRNA or inhibit its translation [20]. Recent studies have demonstrated that MSC-EVs convey neuroprotective and neuroreparative properties through miRNA transport [60]. MiR-486 has been identified as highly abundant in BMSC-EVs [61]. Our prior work demonstrated that miR-486 delivery via BMSC-EVs promoted angiogenesis and functional recovery after cerebral ischemia by suppressing PTEN [23]. Su et al. showed that miR-486-5p in umbilical cord MSC-EVs promoted M1-to-M2 macrophage polarization and protected against diabetic nephropathy [62], while Dori et al. identified miR-486-5p as a regulator of cortical neurogenesis during development [63]. Furthermore, Lang et al. reported that EVs from induced pluripotent stem cells-derived MSCs facilitated neuronal differentiation of hippocampal NSCs via miR-486-5p transfer [64]. Our study demonstrated that TMP preconditioning enriched miR-486 within BMSC-EVs. Critically, administration of TMP-BMSC-EVs significantly elevated miR-486 levels in the peri-infarct region. The essential role of miR-486 was unequivocally demonstrated by the fact that TMP-inhibitor-EVs abolished their therapeutic benefits on functional recovery, M2 polarization, and neurogenesis. These findings indicate that TMP preconditioning exerts its therapeutic effects largely through the upregulation of miR-486 in BMSC-EVs.

Bioinformatic predictions and literature strongly support PTEN as a key target gene of miR-486 [65, 66]. PTEN as a phosphatase enzyme negatively regulates the PI3K/Akt pathway by dephosphorylation of phosphatidylinositol (3,4,5)-trisphosphate (PIP3). PTEN inhibition promotes Akt activation, facilitating the M1-to-M2 shift in microglia after cerebral ischemia-reperfusion injury and traumatic brain injury [67, 68]. Similarly, PTEN knockout promotes M2 polarization and suppresses pro-inflammatory responses post-IS [69]. Furthermore, PTEN is a well-known negative regulator of NSC proliferation, migration, and neuronal differentiation. PTEN inhibitor bpV directly promotes NSC proliferation and neuronal differentiation while inhibiting gliogenesis [70]. Conditional PTEN knockdown enhances neurogenesis in the hippocampus after intracerebral hemorrhage [71], and PTEN deletion improves neuroblast migration towards the ischemic cortex post-stroke [72]. In this study, TMP-BMSC-EVs treatment potently suppressed PTEN and p-NF-κB protein levels while upregulating p-Akt in the peri-infarct area, effects significantly stronger than those of BMSC-EVs. Most importantly, the miR-486 inhibitor abolished the PTEN and p-NF-κB downregulation, p-Akt upregulation, and the associated therapeutic benefits of TMP-BMSC-EVs. Strikingly, these inhibitory effects were rescued by co-administration of the PTEN inhibitor bpV. These results indicate that TMP preconditioning enriches miR-486 in BMSC-EVs, and TMP-BMSC-EVs exert therapeutic effects on IS by transferring miR-486 to inhibit PTEN.

While this study provides novel insights into the therapeutic potential of TMP-BMSC-EVs via the miR-486/PTEN pathway, several limitations warrant mention. First, we did not perform a comprehensive miRNA expression profiling of TMP-BMSC-EVs. Therefore, we cannot exclude the possibility that other miRNAs may also contribute to the observed effects. Second, although PTEN was validated as a key target, miR-486 may regulate other target genes relevant to post-stroke repair, meriting further investigation. Finally, although we demonstrated that TMP-BMSC-EVs promote microglia/macrophage M2 polarization and neurogenesis via miR-486/PTEN pathway, the precise underlying mechanisms remain unclear. Further in vitro studies are needed to elucidate the specific molecular pathways mediating these effects.

## Conclusions

In conclusion, the present study demonstrates that TMP preconditioning enhances the miR-486 content in BMSC-EVs, emphasizing its potential as a treatment strategy for IS. Administration of TMP-BMSC-EVs promoted microglia/macrophages toward to M2 polarization and enhanced neurogenesis by inhibition of PTEN expression through transferring miR-486, thus accelerating neural repair and functional recovery, emerging as a novel therapeutic strategy.

## Supplementary Information

Below is the link to the electronic supplementary material.

Supplementary Material 1

## Data Availability

The data sets used and/or analyzed during the current study are available from the corresponding author upon reasonable request.

## Abbreviations

Akt: Protein kinase B
ANOVA: Analysis of Variance
ALIX: ALG-2-interacting protein X
BCA: Bicinchoninic Acid Assay
BDNF: Brain-derived neurotrophic factor
BM: Bone marrow
BMSC: Bone marrow mesenchymal stem cell
bpV: Bisperoxovanadium
BrdU: 5-bromo-2’-deoxyuridine
CCA: Common carotid artery
CD9: Cluster of differentiation 9
CD63: Cluster of differentiation 63
CD16/32: Cluster of differentiation 16/32
CD206: Cluster of differentiation 206
Cy3: Cyanine 3
DAPI: 4’,6-diamidino-2-phenylindole
DCX: Doublecortin
DMEM/F12: Dulbecco’s Modified Eagle Medium/Nutrient Mixture F-12
ECA: External carotid artery
EV: Extracellular Vesicle
FBS: Fetal bovine serum
FITC: Fluorescein Isothiocyanate
GAPDH: Glyceraldehyde-3-phosphate dehydrogenase
GFAP: Glial fibrillary acidic protein
HS: Hemorrhagic stroke
Iba1: Ionized calcium binding adaptor molecule 1
ICA: Internal carotid artery
IL-1: Interleukin-1
IL-6: Interleukin-6
IL-10: Interleukin-10
IS: Ischemic stroke
MCA: Middle carotid artery
MCAO: Middle cerebral artery occlusion
miR: microRNA
mNSS: Modified neurological severity score
MSC: Mesenchymal stem cell
NC: Negative control
NeuN: Neuronal nuclei antigen
NSC: Neural stem cell
p-Akt: Phosphorylated protein kinase B
PBS: Phosphate-buffered saline
PTEN: Phosphatase and tensin homolog
PVDF: Polyvinylidene fluoride
RIPA: Radioimmunoprecipitation assay
RT-qPCR: Real-time quantitative polymerase chain reaction
SDS-PAGE: Sodium dodecyl sulfate - polyacrylamide gel electrophoresis
SGZ: Subgranular zone
SVZ: Subventricular zone
TEM: Transmission electron microscopy
TGF-β: Transforming growth factor-β
TMP: Tetramethylpyrazine
TNF-α: Tumor necrosis factor-α
VEGF: Vascular endothelial growth factor
