# Supplementary material for: Extracellular vesicles enriched with miR-486 from Tetramethylpyrazine-preconditioned bone marrow mesenchymal stem cells promote microglia/macrophage M2 polarization and enhance neurogenesis in rats with ischemic stroke
Source: Stem Cell Res Ther. 2025 Aug 26;16:455. doi: 10.1186/s13287-025-04574-1 (PMC12382071; doi:10.1186/s13287-025-04574-1)
Supplement: Supplementary file 1 — Supplementary Material 1 [file 13287_2025_4574_MOESM1_ESM.docx]

**Supplementary Figure 1**


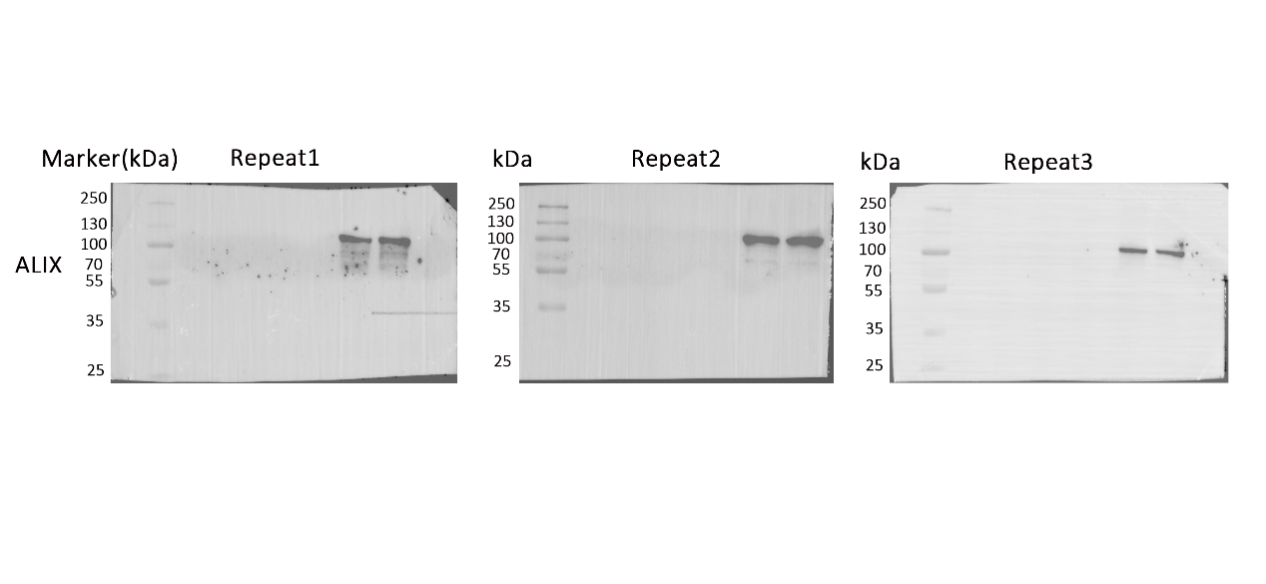


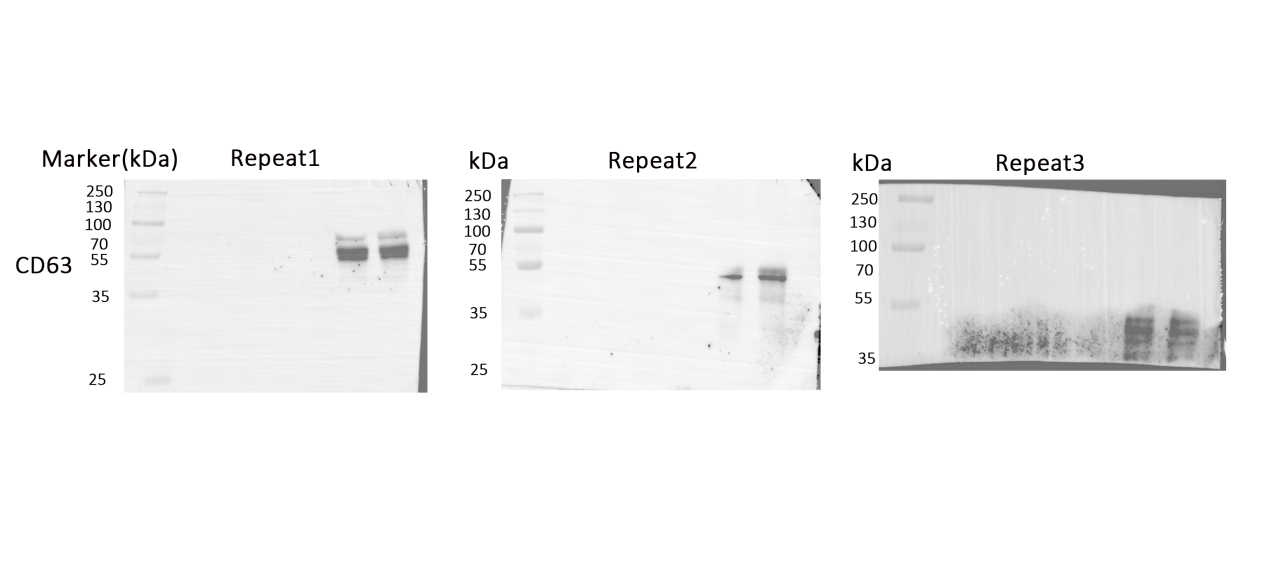


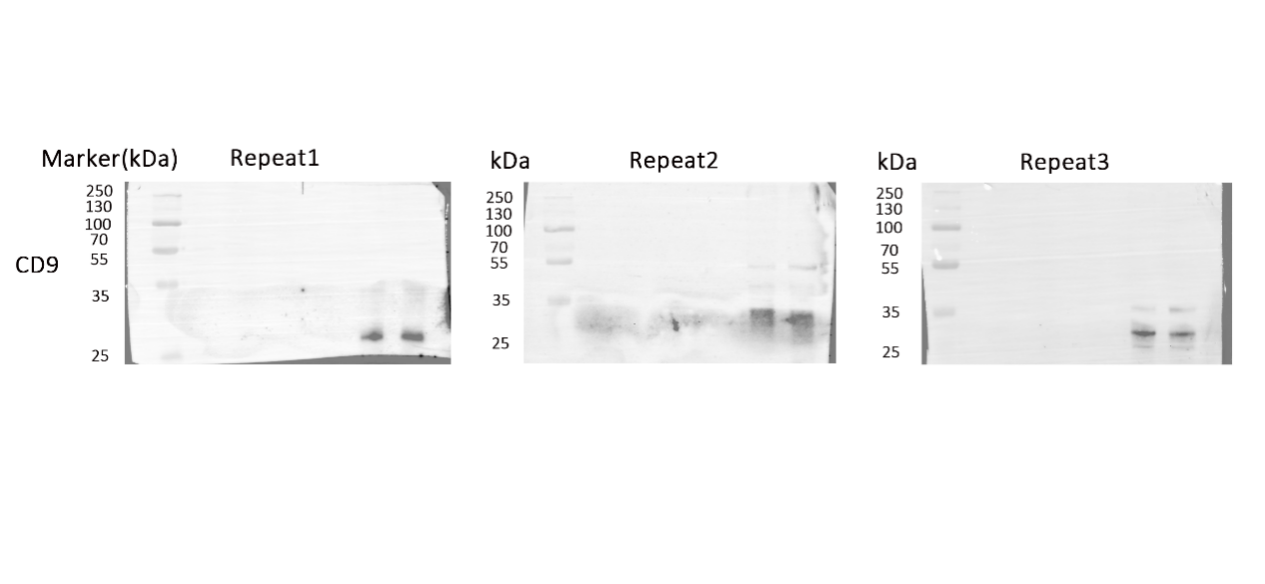


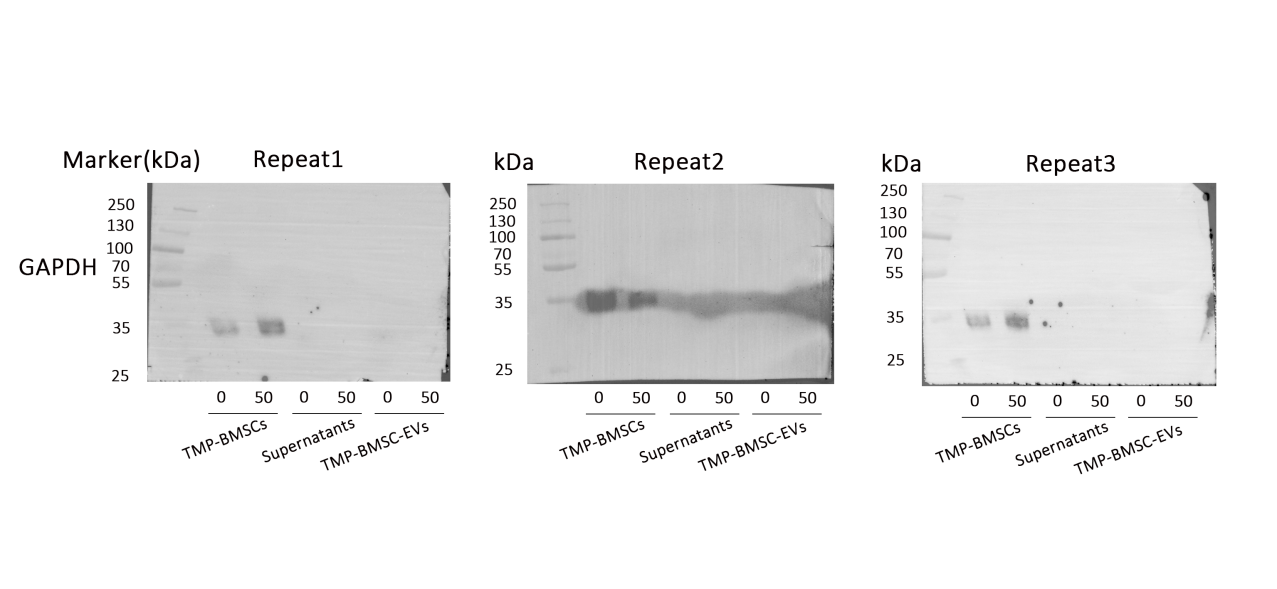


**Supplementary figure 1. Original images of the Western blots showed in Figure 1 C.**

All lane order matches the figures in the manuscript.

**Supplementary Figure 5**

**
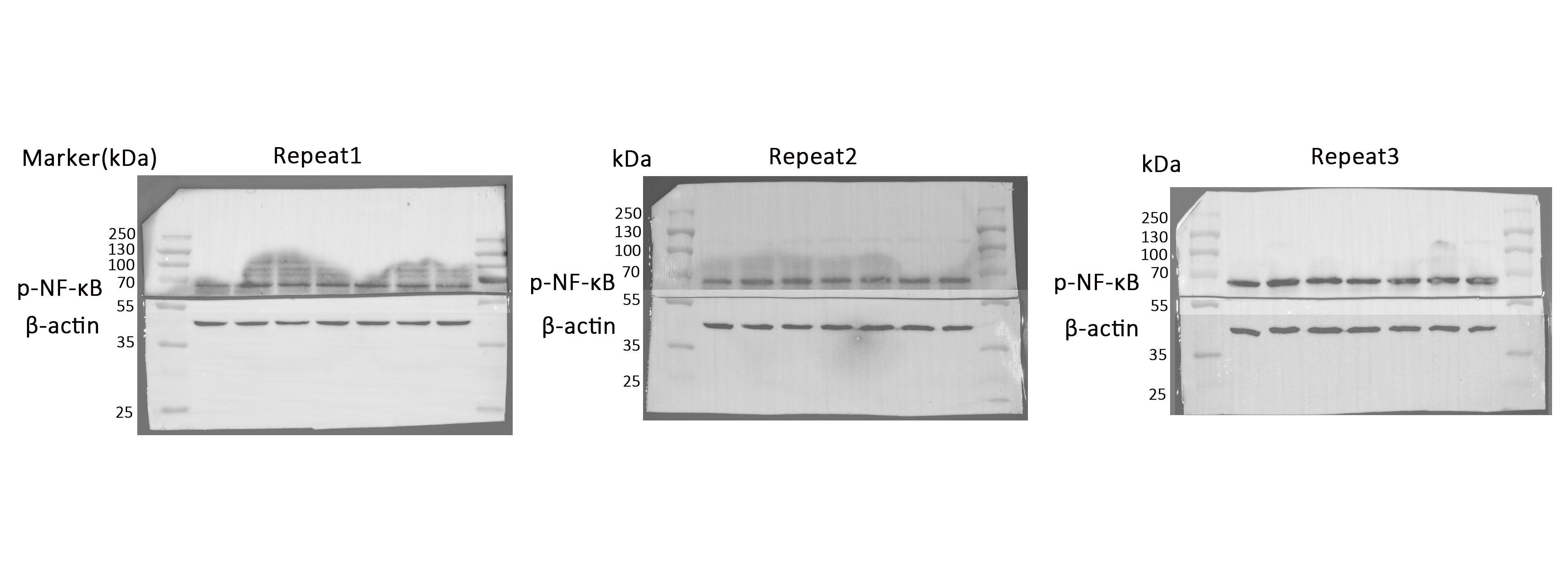
**

**
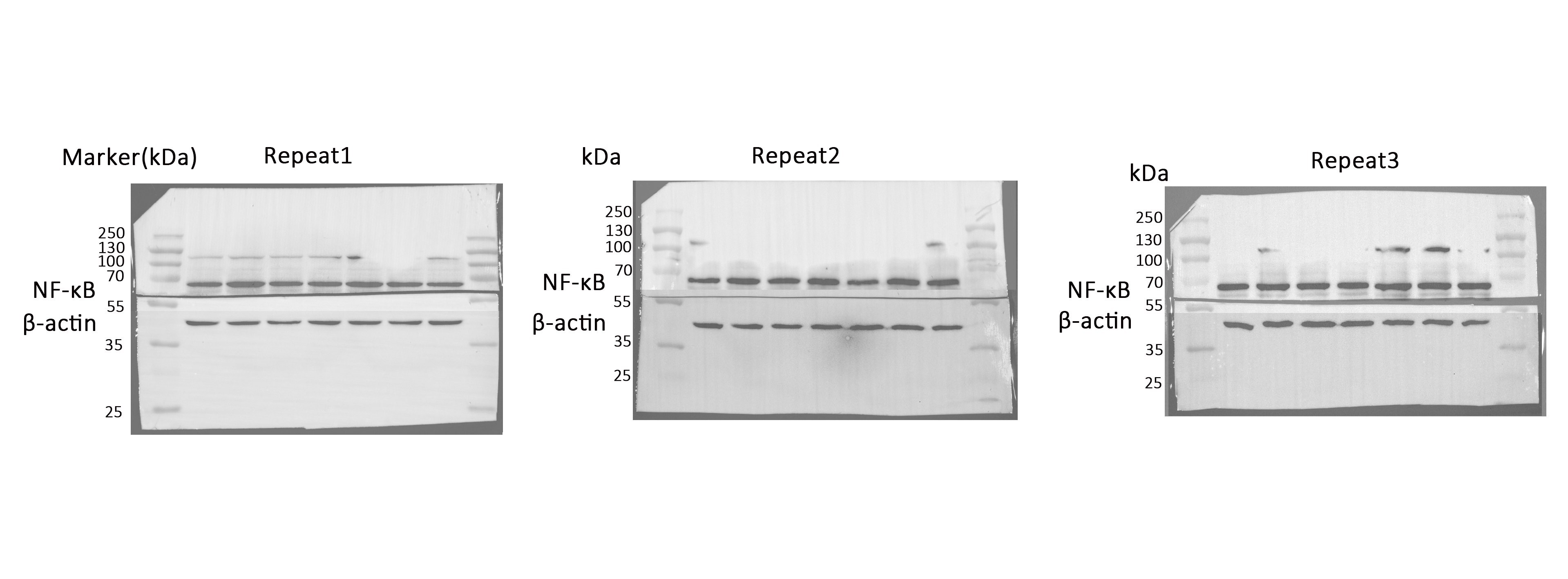
**


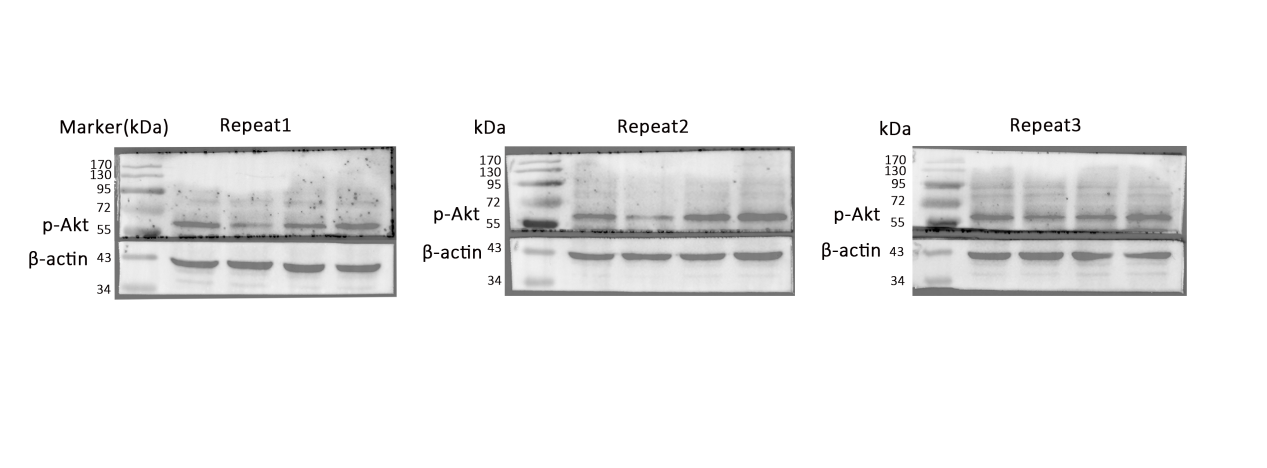


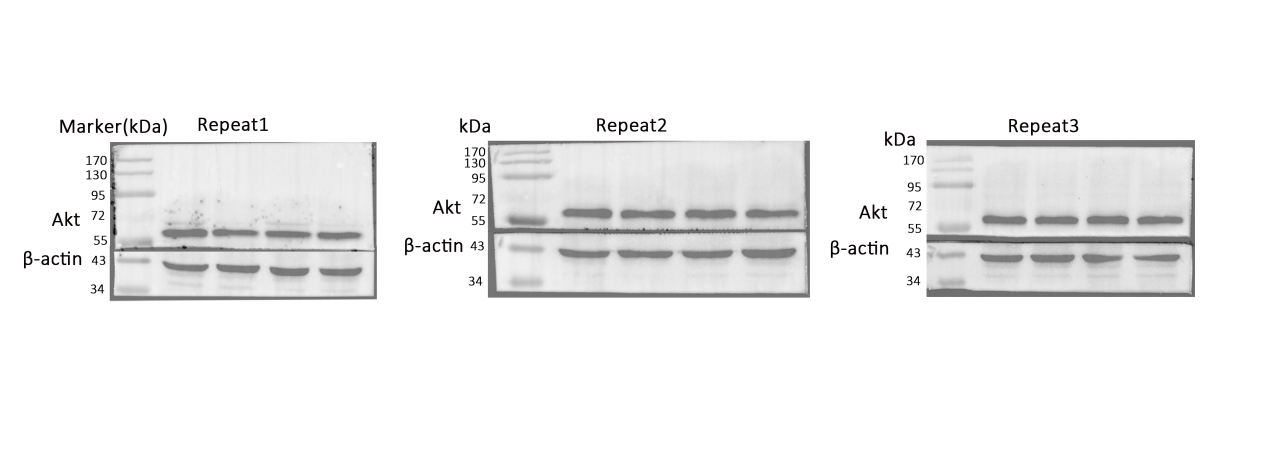


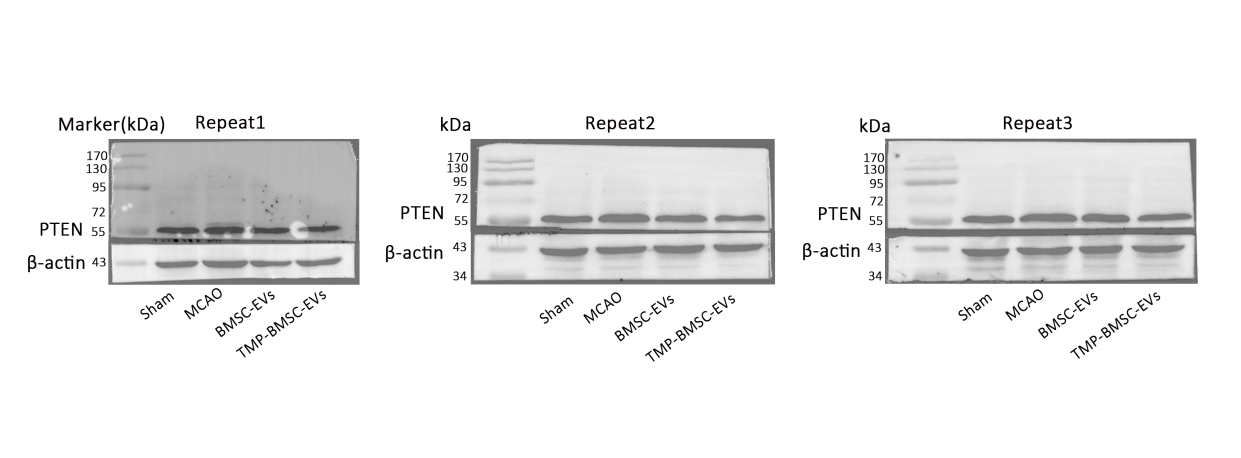


**Supplementary figure 5. Original images of the Western blots showed in Figure 5 C.**

All lane order matches the figures in the manuscript.

**Supplementary Figure 9**

**
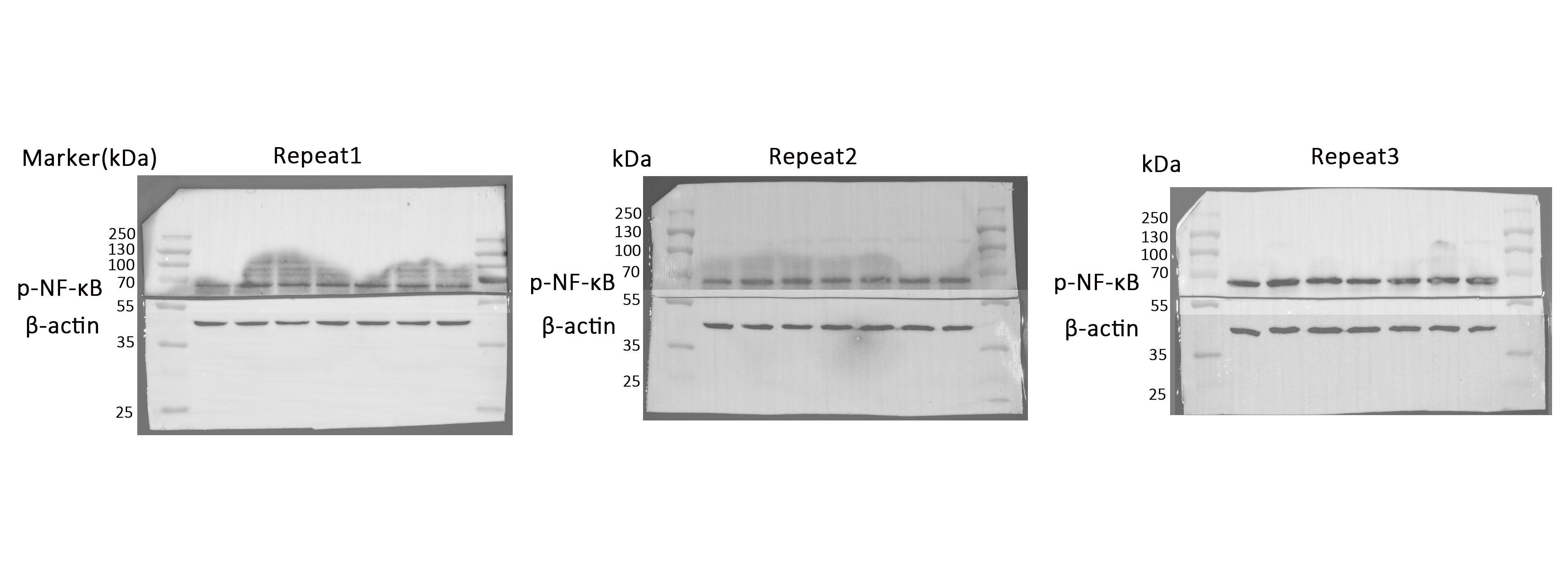
**

**
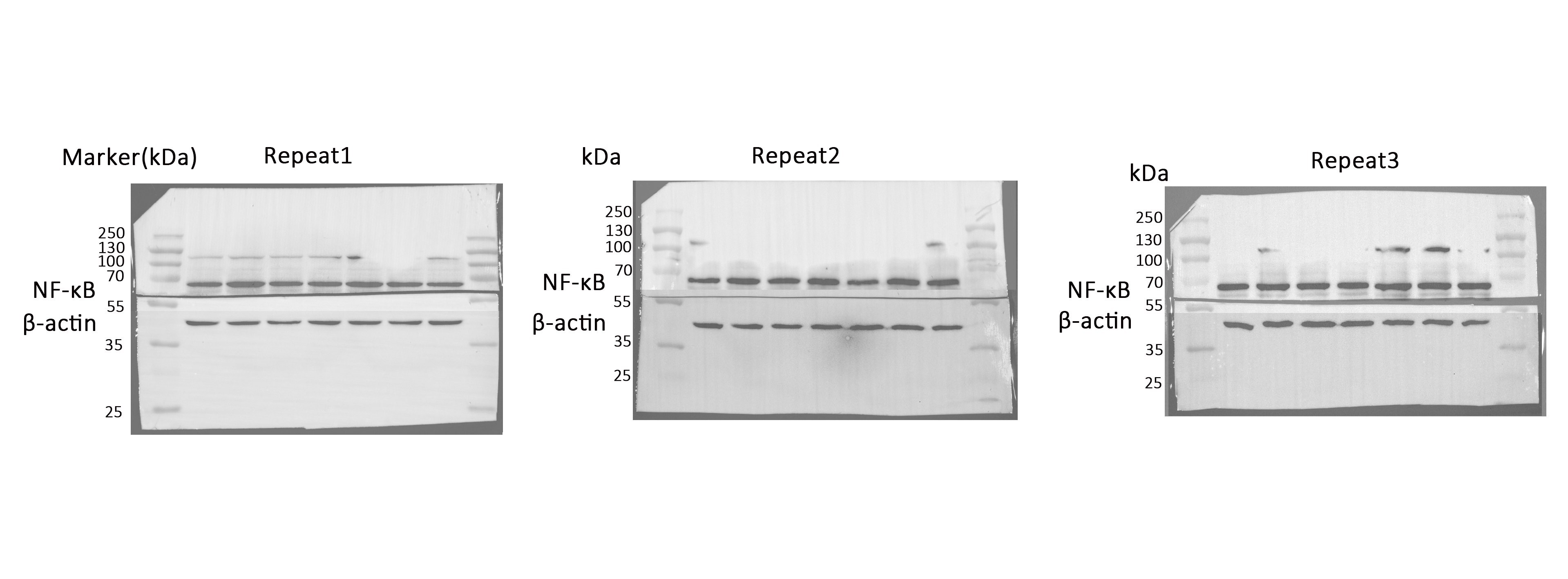
**


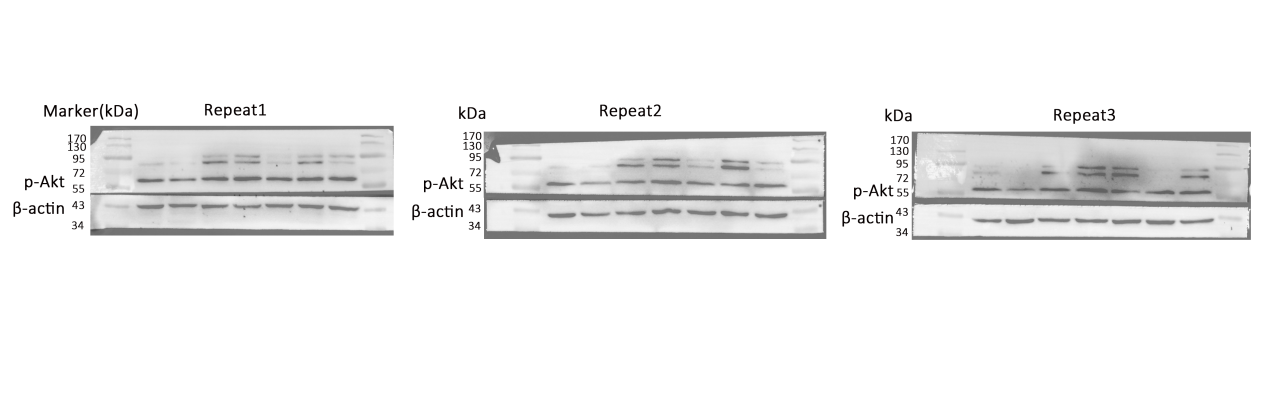


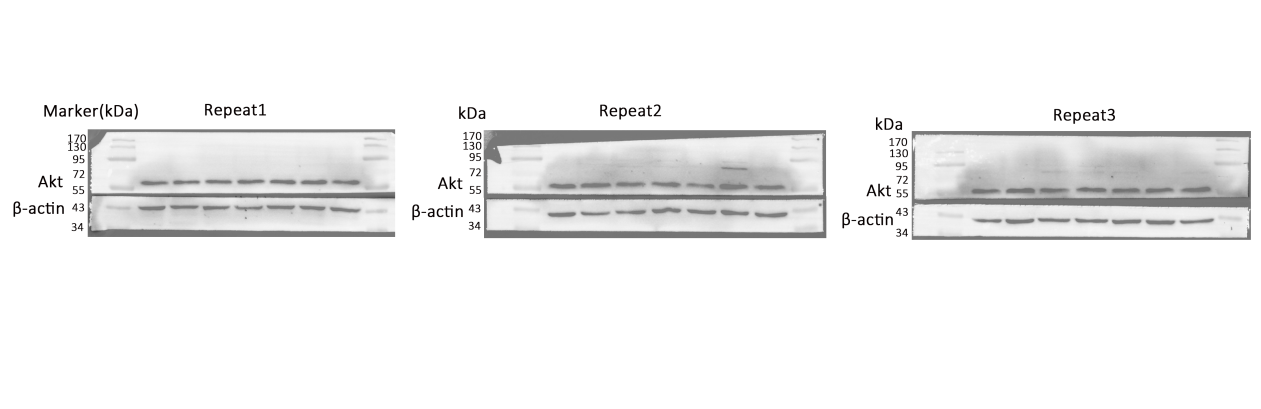


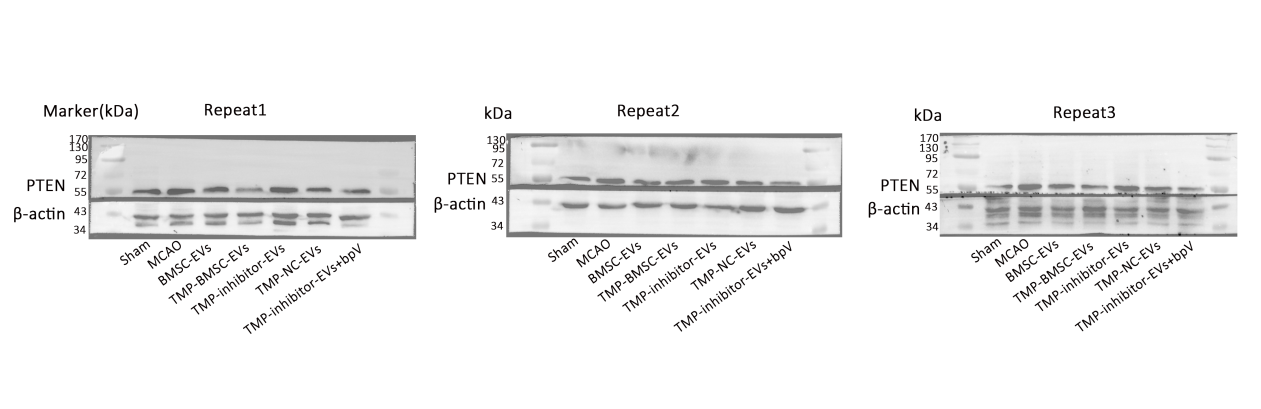


**Supplementary figure 9. Original images of the Western blots showed in Figure 9 B.**

All lane order matches the figures in the manuscript.
